# Hepatic UGT2B-Mediated Testosterone Clearance Promotes Lipid Accumulation in High-Fat-Diet-Induced MASLD

**DOI:** 10.3390/nu18030549

**Published:** 2026-02-06

**Authors:** Liping Zhou, Yingzhuan Zheng, Yujie Qiao, Xin Xu, Duoli Zhang, Yongqiong Shi, Yuanmeng Huang, Hongxiang Zeng, Ting Zeng, Xi Li, Linqiang Zhang

**Affiliations:** 1 Obesity and Metabolic Diseases Research Center, Department of Medical Laboratory Technology, School of Basic Medical Sciences, Chongqing Medical University, Chongqing 400016, China; 2023112100@stu.cqmu.edu.cn (L.Z.); 2024112179@stu.cqmu.edu.cn (Y.Q.); 192298@stu.cqmu.edu.cn (Y.S.); 2023110027@stu.cqmu.edu.cn (Y.H.); summerbreeze@stu.cqmu.edu.cn (H.Z.); zengting@cqmu.edu.cn (T.Z.); 2 Basic Medicine Research and Innovation Center for Novel Target and Therapeutic Intervention (Ministry of Education), College of Pharmacy, Chongqing Medical University, Chongqing 400016, China; 191552@cqmu.edu.cn; 3 Molecular Medical Laboratory, School of Basic Medical Sciences, Chongqing Medical University, Chongqing 400016, China

**Keywords:** MASLD, testosterone, UGT2B, cholesterol, high-fat diet

## Abstract

**Background and Objective**: Male individuals diagnosed with metabolic dysfunction-associated steatotic liver disease (MASLD) frequently present with decreased blood testosterone concentrations concomitant with increased levels of hepatic cholesterol, the fundamental substrate for testosterone synthesis; however, the mechanistic relationship between these phenomena remains inadequately elucidated. This study aimed to examine the involvement of hepatic cholesterol biosynthesis and testosterone metabolism in the pathogenesis of MASLD. **Methods**: An MASLD model was established in male C57BL/6J mice subjected to a high-fat diet (HFD). Comprehensive analyses, including hepatic transcriptomics, metabolomics, enzyme-linked immunosorbent assay, Western blotting, and quantitative polymerase chain reaction, were conducted. Additionally, in vitro experiments were performed using AML-12 hepatocytes treated with oleic acid and testosterone, with or without the presence of a uridine diphosphate-glucuronosyltransferase family 2 member B (UGT2B) enzyme inhibitor. **Results**: The HFD elevated cholesterol levels and activated cholesterol synthesis and testosterone metabolic pathways, notably characterized by upregulation of UGT2B enzymes and their transcriptional regulator, the aryl hydrocarbon receptor (AHR). Blood testosterone increased initially but decreased after 24 weeks of HFD. In vitro, testosterone alone did not affect oleic acid-induced lipid accumulation, but inhibiting UGT2B enabled testosterone levels to reduce lipid deposition and downregulate lipid uptake and synthesis pathways. **Conclusions**: The HFD induces dynamic, UGT2B-mediated hepatic testosterone metabolism. Compensatory early testosterone increase is offset by enhanced UGT2B-mediated clearance, resulting in eventual testosterone depletion and the loss of its protective effects against hepatic lipid accumulation. This explains the clinical paradox and suggests targeting the hepatic UGT2B enzymes as a potential MASLD treatment.

## 1. Introduction

Metabolic dysfunction-associated steatotic liver disease (MASLD) represents one of the most prevalent chronic liver disorders globally, impacting approximately 38% of the adult population worldwide [1]. The hallmark pathological characteristic of MASLD is the excessive intracellular accumulation of triglycerides within hepatocytes, a condition frequently linked to obesity, insulin resistance, and metabolic syndrome. The disease trajectory encompasses a spectrum of stages, including simple steatosis, metabolic dysfunction-associated steatohepatitis (MASH), fibrosis, cirrhosis, and ultimately, hepatocellular carcinoma [2]. Despite its significant public health implications and substantial societal burden [3], the precise molecular mechanisms underlying MASLD progression remain inadequately elucidated, thereby impeding the development of effective therapies.

In recent years, growing interest has focused on the contribution of endocrine hormone imbalances to the initiation and progression of MASLD [4,5]. These imbalances include deficiencies in growth, sex, and thyroid hormones, as well as hypercortisolemia. Accumulating evidence indicates that disruptions in sex hormone metabolism may play a critical role in the pathogenesis and advancement of MASLD [6,7,8,9]. Among these hormones, androgens, particularly testosterone, have emerged as a focal point in investigations concerning MASLD in male populations. Numerous cross-sectional and longitudinal cohort studies have consistently shown that circulating testosterone levels are significantly lower in male patients with MASLD compared to healthy individuals [10,11,12,13,14,15]. Moreover, testosterone concentrations tend to decline progressively with disease severity, from simple steatosis through MASH to fibrosis [16]. These associations suggest a potential hepatoprotective role of testosterone. Nevertheless, the mechanisms by which testosterone confers hepatic protection, as well as the factors contributing to its diminished levels in MASLD, require further comprehensive investigation.

MASLD is frequently linked to obesity and metabolic disorders, which are characterized by elevated hepatic cholesterol content [17]. Such elevated liver cholesterol has also been observed in murine models of MASLD induced by high-fat diet (HFD) [18]. Cholesterol serves not only as an essential structural component of cellular membranes but also as the universal precursor for the biosynthesis of all steroid hormones, including testosterone [19]. These observations indicate that the liver in MASLD may experience a state of “cholesterol surplus” alongside an abundance of substrates for steroidogenesis. Paradoxically, however, systemic testosterone levels are often reduced under these conditions, and the underlying regulatory mechanisms remain poorly understood. In obese males presenting with hypogonadism, prevailing hypotheses attribute low testosterone levels primarily to the suppression of the hypothalamic–pituitary–gonadal (HPG) axis. According to this view, adipocytes indirectly inhibit testosterone production by secreting signaling molecules such as leptin and pro-inflammatory cytokines [20,21,22]. Despite this, the liver’s role—as a critical target organ and a principal site for testosterone metabolism and clearance—has been less explored regarding its direct influence on testosterone bioavailability in the context of MASLD.

The uridine diphosphate-glucuronosyltransferase (UGT) enzyme family in the liver, particularly the UGT2B subfamily, plays a critical role in the glucuronidation-mediated metabolism of testosterone. This metabolic pathway constitutes the principal mechanism for testosterone inactivation and clearance in the liver, thereby promoting its elimination via urinary and biliary excretion [23]. In the human liver, the predominant UGT2B isoforms include UGT2B4, UGT2B7, UGT2B10, UGT2B11, UGT2B15, and UGT2B17 [24]. Conversely, in the murine liver, the principal Ugt2b isoforms comprise Ugt2b1, Ugt2b5, Ugt2b34, Ugt2b35, Ugt2b36, Ugt2b37, and Ugt2b38 [25]. The transcriptional regulation of these enzymes is modulated by multiple transcription factors. Notably, the aryl hydrocarbon receptor (AHR) is a pivotal regulator in the metabolism of both exogenous and endogenous compounds and has been demonstrated to upregulate UGT2B gene expression [26,27]. Moreover, studies indicate that the genetic or pharmacological inhibition of AHR signaling mitigates HFD-induced pathologies, including obesity, hepatic lipid accumulation, glucose intolerance, and insulin resistance [28,29]. Nonetheless, current research has yet to elucidate whether AHR modulates lipid accumulation during MASLD development through its regulatory effects on testosterone metabolism.

Current studies indicate that the suppression of the HPG axis via adipose tissue-derived factors contributes to hypogonadism in MASLD; however, this perspective overlooks the liver’s active metabolic role. The AHR is well-established for its roles in xenobiotic metabolism and the regulation of lipid metabolism in obesity and MASLD [30,31,32]. Similarly, UGT2B enzymes are known for drug metabolism functions, such as morphine glucuronidation [33]. Nevertheless, a potential liver-intrinsic pathway involving the concerted action of AHR and UGT2B in promoting testosterone clearance in MASLD remains to be elucidated. We propose that diet-induced activation of AHR upregulates hepatic UGT2B expression, thereby facilitating enhanced local inactivation of testosterone.

This study elucidates a novel pathogenic mechanism in MASLD. We demonstrate that short-term HFD exposure increases circulating testosterone levels through enhanced hepatic steroidogenesis. In contrast, prolonged HFD induces the AHR and UGT2B, which accelerate hepatic testosterone clearance. This enhanced clearance results in reduced circulating testosterone levels, thereby obscuring testosterone’s inherent hepatoprotective effects and exacerbating MASLD. Notably, the inhibition of UGT2B restores testosterone’s capacity to mitigate lipid accumulation by downregulating CD36 and DGAT2 expression. These results identify UGT2B-mediated testosterone clearance as a critical factor and suggest that it conceals testosterone’s intrinsic protective role in MASLD. Collectively, our findings advance the understanding of the interplay between hormonal regulation and metabolic dysfunction in MASLD in male patients. They also highlight hepatic testosterone clearance as a potential therapeutic target.

## 2. Materials and Methods

### 2.1. Epidemiologic Analysis of Relationship Between Blood Testosterone Levels and MASLD in NHANES Data

Data from the 2013–2016 cycle of the National Health and Nutrition Examination Survey (NHANES) were obtained from the official database at https://wwwn.cdc.gov/nchs/nhanes/ (accessed on 24 November 2025). From an initial pool of 20,146 participants, we excluded 4441 individuals with excessive alcohol consumption (defined as ≥30 g/day for men and ≥20 g/day for women), leaving 15,705 participants. Among these, 11,854 individuals had complete data on triglyceride (TG), body mass index (BMI), gamma-glutamyl transferase (GGT), and waist circumference (WC), and they were included for further analysis. We then excluded 6610 participants aged < 18 or >60 years, resulting in a final analytical cohort of 5224 participants (2450 males and 2794 females).

MASLD was defined using three validated noninvasive indices—the Hepatic Steatosis Index (HSI), the Fatty Liver Index (FLI), and the NAFLD Liver Fat Score (NAFLD-LFS)—with established formulas and diagnostic cut-offs applied as described previously [34]. Specifically, they are as follows:

HIS = 8 × AST/ALT + BMI (+2 if diabetes mellitus, +2 if female).

FLI = [e^ (0.953 × ln (TG) + 0.139 × BMI + 0.718 × ln (GGT) + 0.053 × WC − 15.745)]/[1 + e^ (0.953 × ln (TG) + 0.139 × BMI + 0.718 × ln (GGT) + 0.053 × WC − 15.745)] × 100.

NAFLD-LFS = −2.89 + 1.18 × metabolic syndrome (yes = 1/no = 0) + 0.45 × T2DM (yes = 2/no = 0) + 0.15 × fasting serum insulin (uU/mL) + 0.04 × AST (U/L) − 0.94 × AST/ALT.

Participants were classified as having MASLD if HSI > 36, FLI > 60, or NAFLD-LFS > −0.640.

To assess the association between sex hormones and MASLD, we stratified participants by sex and categorized hormone levels into sex-specific quartiles (Q1–Q4). For men, testosterone and sex-hormone-binding globulin (SHBG) were analyzed; for women, testosterone, SHBG, and free testosterone were analyzed. The lowest quartile served as the reference. Hierarchical multivariable logistic regression was performed using three sequentially adjusted models:

Model 1: Age and alcohol consumption.
Model 2: Model 1 plus insulin resistance (HOMA-IR), smoking status, and medication use.
Model 3: Model 2 plus BMI.

Odds ratios (ORs) and 95% confidence intervals (CIs) were estimated. A complete-case analysis was applied, excluding participants with missing data for any variable in the fully adjusted model.

Blood samples in the NHANES cycles were collected through standardized morning draws to minimize diurnal variation. Serum testosterone was measured using isotope-dilution liquid chromatography–tandem mass spectrometry (ID-LC-MS/MS), with results reported in ng/dL and a lower limit of detection of 0.75 ng/dL. Detailed assay methodology is available on the NHANES website at https://wwwn.cdc.gov/Nchs/Data/Nhanes/Public/2013/DataFiles/TST_H.htm (accessed on 24 November 2025) and https://wwwn.cdc.gov/Nchs/Data/Nhanes/Public/2015/DataFiles/TST_I.htm (accessed on 24 November 2025).

### 2.2. Mouse Husbandry and Experimentation

To establish an MASLD model, 80 male C57BL/6J mice aged 8 weeks were randomly assigned to two experimental groups: a control group (Control) fed a standard chow diet and a high-fat-diet group (HFD) provided with a diet comprising 60% of calories from fat (D12492; Research Diets). MASLD progression was evaluated at 4, 8, 16, and 24 weeks after diet initiation, with 10 mice per group evaluated at each time point. All animals were purchased from and maintained at the Laboratory Animal Research Center of Chongqing Medical University under specific pathogen-free (SPF) conditions. They were maintained under a 12 h light/dark cycle at a controlled temperature of 25 ± 1 °C, with ad libitum access to food and water. At the designated time point, the mice were euthanized, followed by tissue collection. Blood samples were collected and then centrifuged at 3000 rpm for 5 min. The resulting plasma was stored at −80 °C prior to analysis. Liver specimens were either fixed in 10% formalin for histological examination or rapidly frozen in liquid nitrogen and stored at −80 °C for subsequent analyses. All animal experiments were approved by the Institutional Animal Care and Use Committee of Chongqing Medical University (No. IACUC-CQMU-2025-0231).

### 2.3. Glucose and Insulin Tolerance Test

A glucose tolerance test (GTT) was conducted on the Control and HFD mice following an overnight fast, utilizing the intraperitoneal administration of glucose at a dosage of 2 g/kg body weight. Insulin tolerance test (ITT) was performed on Control and HFD mice after 4 h of food deprivation via an intraperitoneal injection of insulin at a dosage of 0.75 U/kg body weight. In both tests, blood glucose levels were measured from tail blood samples prior to and at specified intervals following the injections.

### 2.4. Histologic Analysis

For hematoxylin and eosin (H&E) staining, liver specimens were promptly fixed in 10% neutral-buffered formalin after harvesting, subsequently embedded in paraffin, sectioned at a thickness of 5 μm, and stained with hematoxylin and eosin according to the standard protocols. For Oil Red O (ORO) staining, liver samples were initially embedded in an optimal cutting temperature (OCT) compound and subsequently sectioned at a thickness of 30 μm. The sections were then stained with ORO to detect neutral lipids, which appear red, followed by counterstaining with hematoxylin to visualize nuclei, which are stained blue.

### 2.5. Cell Culture and Lipid Droplet Staining

The AML-12 mouse hepatocyte cell line was maintained in Dulbecco’s Modified Eagle Medium (C11995500CPGibco, Grand Island, NE, USA) supplemented with 10% fetal bovine serum (10099-141, Gibco, Grand Island, NE, USA) and 100 U/mL penicillin-streptomycin. The cell cultures were incubated at 37 °C in a humidified atmosphere containing 5% CO_2_.

To induce lipid accumulation, oleic acid (OA) (O3880, Sigma-Aldrich, St. Louis, MO, USA) was complexed with 10% (*w*/*v*) fatty acid-free bovine serum albumin (BSA) in PBS under gentle heating (37 °C) and vortexing. Cells were then exposed to this OA-BSA complex at a final OA concentration of 0.2 mM for 24 h. To assess the potential inhibitory effect of testosterone on lipid accumulation, cells were first treated with 30 µM of probenecid (T0457, TargetMol, Shanghai, China; a UGT2B inhibitor, termed Ui) together with OA for 12 h. Then, 1 µM testosterone (Zhiyi Biotech, Taizhou, China) was added, and the cells were incubated for another 12 h in the continued presence of OA and Ui.

For ORO staining, cells were fixed in 4% paraformaldehyde at 4 °C for 15 min and then incubated with the ORO working solution—prepared by diluting a 5 mg/mL ORO stock solution with distilled water at a 3:2 ratio—for 2 h. Following thorough washing, nuclear counterstaining was performed using hematoxylin. Subsequently, the samples were mounted with glycerol gelatin and examined via light microscopy.

For Bodipy staining, cells were fixed with 4% paraformaldehyde at 4 °C for 15 min and then stained with 5 µM Bodipy dye (D3922, ThermoFisher, Waltham, MA, USA) at 37 °C for 30 min. After washing, nuclei were counterstained with DAPI. Finally, the specimens were mounted using an anti-fade mounting medium and visualized under a confocal microscope.

### 2.6. Total RNA Extraction and qPCR

Total RNA extraction and quantitative PCR (qPCR) were conducted following the methodology previously described by Zeng et al. [35]. Briefly, total RNA was extracted using a TRIzol reagent (ThermoFisher, Carlsbad, CA, USA), and 1 μg of RNA was reverse-transcribed into cDNA using a commercial kit. Quantitative real-time PCR was performed with SYBR Green Master Mix on an ABI Prism 7500 system. Gene expression levels were normalized to β-actin and analyzed using the 2^−ΔΔCt^ method. The list of primers used in this study can be found in Table S1.

### 2.7. Protein Extraction and Western Blot Analysis

Protein extraction and Western blot (WB) analysis were conducted following the methodology previously described by Zhang et al. [36]. Briefly, proteins were extracted from liver tissues or cultured cells using a lysis buffer supplemented with protease and phosphatase inhibitors. Protein concentrations were determined via the BCA method. After denaturation, proteins were separated by SDS-PAGE and transferred onto PVDF membranes. The membranes were blocked and then incubated with specific primary antibodies overnight at 4 °C. Following primary antibody incubation, the membranes were washed and incubated with HRP-conjugated secondary antibodies for 1 h at room temperature. After final washes, protein bands were visualized using enhanced chemiluminescence and detected with a chemiluminescence imaging system. The list of antibodies used in this study can be found in Table S2.

### 2.8. Mouse Liver Transcriptome Analysis

Transcriptome sequencing and analysis were performed by OE Biotech Co., Ltd. (Shanghai, China). Initially, the total RNA was extracted from mouse liver tissue, followed by quantification and quality assessment. A total amount of 1.5 μg RNA per sample was used as input material for the RNA sample preparations. Subsequently, Sequencing libraries were constructed using the NEBNext^®^ UltraTM RNA Library Prep Kit for Illumina^®^ (NEB, Ipswich, MA, USA) according to the manufacturer’s protocol. Briefly, mRNA was enriched, fragmented, and reverse-transcribed into cDNA. The cDNA fragments underwent end repair, adenylation, and adapter ligation, followed by size selection and PCR amplification. Library quality was assessed using an Agilent Bioanalyzer 2100 system (Santa Clara, CA, USA). Finally, paired-end sequencing (150 bp) was performed on an Illumina HiSeq 4000 platform (San Diego, CA, USA).

Raw reads were processed with fastp (v0.18.0) to remove adapters and low-quality bases, yielding clean reads. Ribosomal RNA reads were identified and removed by alignment to an rRNA database using Bowtie2 (v2.2.8). The remaining reads were mapped to the reference genome (GRCm38/mm10) using HISAT2 (v2.1.0). Transcript assembly and expression quantification (in FPKM) were performed with StringTie (v1.3.1) and RSEM, respectively.

Differential expression analysis was performed using DESeq2. Genes with a false discovery rate (FDR) of <0.05 and an absolute fold change of ≥1.5 were defined as differentially expressed genes (DEGs). Functional enrichment analysis of DEGs was carried out based on the GO and KEGG databases. The calculated *p*-value underwent FDR correction, using a q-value of <0.05 as a threshold. For gene set enrichment analysis, the gene expression matrix was analyzed using GSEA, and normalized enrichment scores, *p*-value, and adjusted *p*-value were calculated with default parameters. Significantly enriched pathways were defined as those with an |NES| > 1.5 and an FDR q-value < 0.05. All bioinformatics analyses were conducted using the OECloud tools at https://cloud.oebiotech.com.

### 2.9. Mouse Liver Metabolomics Analysis

Metabolomic profiling was performed by OE Biotech Co., Ltd. (Shanghai, China). In brief, 30 mg of liver tissue was homogenized with steel beads using 400 μL of pre-cooled methanol–water solution (4:1, *v*/*v*) containing L-2-chlorophenylalanine at a concentration of 4 μg/mL. Then, the homogenate was ultrasonicated in an ice-water bath and incubated at −40 °C. After centrifugation, the supernatant was collected and evaporated to dryness, and the residue was reconstituted in methanol–water (1:4, *v*/*v*). The extract was again incubated at −40 °C to precipitate proteins, followed by a second centrifugation. The final supernatant was filtered through a 0.22 μm membrane for LC-MS analysis. A pooled quality control (QC) sample was prepared by combining equal volumes of all individual extracts. All steps were performed under cold conditions to maintain metabolite stability.

LC-MS analysis was conducted using an ACQUITY UPLC I-Class Plus system coupled with a high-resolution mass spectrometer. Chromatographic separation was performed on an ACQUITY UPLC HSS T3 column (100 mm × 2.1 mm, 1.8 μm particle size) maintained at 45 °C. The mobile phase comprised solvent A (water with 0.1% formic acid) and solvent B (acetonitrile), delivered at a flow rate of 0.35 mL/min. An injection volume of 5 μL was applied for all samples.

The original LC-MS data were processed using Progenesis QI V2.3 (Nonlinear, Dynamics, Newcastle, UK) for baseline filtering, peak identification, integration, retention time correction, peak alignment, and normalization. The main parameters—5 ppm precursor tolerance, 10 ppm product tolerance, and 5% product ion threshold—were applied. Compound identification was carried out based on precise mass-to-charge ratios (*m*/*z*), secondary fragments, and isotopic distributions using the Human Metabolome Database (HMDB), Lipidmaps (V2.3), Metlin, EMDB, PMDB, and self-built databases in order to carry out qualitative analysis.

The extracted data were then further processed by removing any peaks with a missing value (ion intensity = 0) in more than 50% of the group, replacing zero values with half of the minimum value, and carrying out screening according to the qualitative results of the compounds. Compounds with resulting scores below 36 (out of 60) points were also deemed to be inaccurate and removed. A data matrix was combined from the positive and negative ion data.

The matrix was imported into R to carry out principal component analysis (PCA) in order to observe the overall distribution among the samples and the stability of the whole analysis process. Orthogonal partial least squares–discriminant analysis (OPLS-DA) was utilized to distinguish the metabolites that differ between groups. To prevent overfitting, 7-fold cross-validation and 200 response permutation testing (RPT) were used to evaluate the quality of the model.

Variable importance of projection (VIP) values obtained from the OPLS-DA model were used to rank the overall contribution of each variable to group discrimination. A two-tailed Student’s *t*-test was further used to verify whether the metabolites of difference between groups were significant. Differential metabolites were selected, with VIP values greater than 1.0 and *p*-values of less than 0.05.

### 2.10. Plasma and Liver Lipid Measurement

Plasma and liver lipids, such as triglyceride (TG), total cholesterol (TC), free cholesterol (FC), high-density lipoprotein cholesterol (HDL-c), and low-density lipoprotein cholesterol (LDL-c), were measured using commercial kits (Nanjing Jiancheng, Nanjing, China and Solarbio, Beijing, China) according to the manufacturer’s protocols.

### 2.11. Plasma LH, FSH, TT, and T-Gluc Concentration Determination

Mouse plasma concentrations of luteinizing hormone (LH), follicle-stimulating hormone (FSH), testosterone (TT), and testosterone glucuronide (T-Gluc) were detected using specific commercially available enzyme-linked immunosorbent assay (ELISA) kits (Shanghai Yuanxin Biotech, Shanghai, China), in accordance with the manufacturer’s instructions. Each analyte was quantified in a separate assay using its dedicated kit, following a common principle and procedure.

Briefly, the samples and serially diluted standards of known concentration were added in duplicate to microplate wells pre-coated with a capture antibody specific to the target analyte (i.e., anti-LH, anti-FSH, anti-T, or anti-T-Gluc antibody, depending on the kit). Subsequently, an HRP-conjugated detection antibody specific to the same target was added to each well. After incubation and thorough washing, the plates were incubated with the chromogenic substrate 3,3′,5,5′-tetramethylbenzidine (TMB). The enzymatic reaction catalyzed by HRP produced a blue product, which was converted to a stable yellow solution after adding a stop solution. The intensity of the yellow color, measured as the OD at 450 nm using a microplate reader, was directly proportional to the concentration of the target analyte in the sample. The concentration was calculated based on the standard curve.

The concentrations of LH, FSH, TT, and T-Gluc are expressed in mU/mL, mIU/mL, pg/mL, and pg/mL, respectively. The minimum detectable concentrations for these assays were 0.1 mU/mL for LH, 1.0 mIU/mL for FSH, 1.0 pg/mL for TT, and 10.0 pg/mL for T-Gluc.

### 2.12. Hepatic UGT2B Activity Determination

The enzyme activity of UGT2B was determined using a commercial ELISA kit (Shanghai Yuanxin Biotech, Shanghai, China). The assay procedure was performed as follows. Prior to the immunoassay, samples were subjected to an enzymatic reaction pretreatment to activate the antigenic epitopes. Specifically, a UGT2B-specific reaction mixture containing the glucuronic acid donor UDPGA and a specific aglycone acceptor substrate was added to the samples. Following incubation at the recommended temperature, UGT2B present in the samples catalyzed the glucuronidation of the substrate.

Subsequently, the pretreated samples, alongside a series of standard solutions with known concentrations, were added to microplate wells pre-coated with a capture antibody specific to UGT2B. After incubation and washing, an HRP-conjugated detection antibody was added to the wells and allowed to bind. Following another incubation and thorough washing step, the chromogenic substrate TMB was added. TMB was catalyzed by HRP to produce a blue color, which was then converted into a stable yellow solution after the addition of a stop solution (acid). The intensity of the developed yellow color, measured as the OD at 450 nm using a microplate reader, was directly proportional to the amount of catalytically active UGT2B present in the sample. The enzyme activity of UGT2B in the samples was calculated by interpolating the OD values against the standard curve. The activity of UGT2B was measured in units per liter (U/L), with an assay sensitivity (limit of detection) of 0.1 U/L.

### 2.13. Statistical Analyses

Data are presented as mean ± SD. For comparisons between two groups, the unpaired two-tailed Student’s *t*-test was used. For comparisons among five groups, one-way analysis of variance (ANOVA) was performed, followed by Tukey’s HSD multiple comparison. Variables were tested for normality using a Shapiro–Wilk test. Pearson correlation analysis was employed to examine the relationship between the two variables presented in the figures. *p* < 0.05 was considered statistically significant. All statistical comparisons were performed using SPSS 20.0. All graphs were produced using Origin 2024. Schematic diagrams and figure layouts were generated using Adobe Illustrator 2023.

## 3. Results

### 3.1. Analysis of Population Data Reveals Markedly Reduced Blood Testosterone Levels in Male Patients with MASLD Compared to the Normal Population

**Figure 1:**
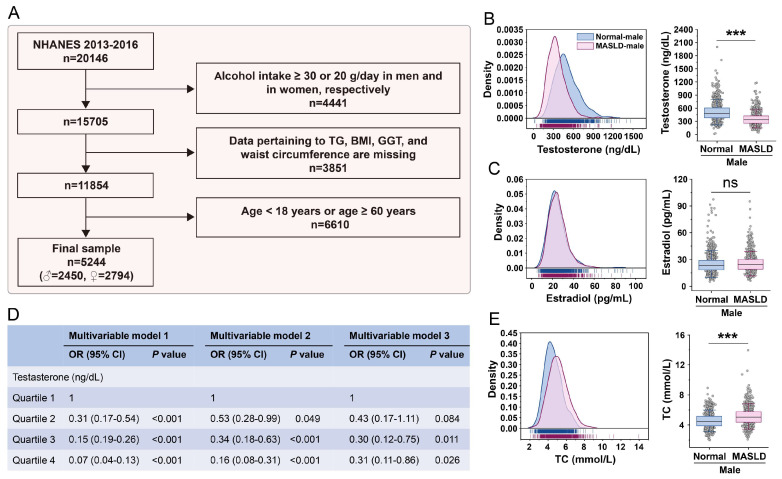
Acquisition of NHANES data and evaluation of associated parameters within the male cohort. (**A**) Inclusion and exclusion criteria applied to the dataset. (**B**) Changes in blood testosterone levels in males with MASLD. (**C**) Changes in blood estradiol levels in males with MASLD. (**D**) Association between testosterone concentrations and MASLD as defined by the Hepatic Steatosis Index (HSI) in men. Multivariable model 1 was adjusted for age and alcohol consumption. Multivariable model 2 was adjusted for age, alcohol consumption, insulin resistance (HOMA-IR), smoking status, and medication use. Multivariable model 3 includes BMI in addition to the variables addressed in model 2. Testosterone: Quartile 1, <348.00 ng/dL; Quartile 2, 348.00–452.50 ng/dL; Quartile 3, 452.50–556.47 ng/dL; Quartile 4, ≥556.47 ng/dL. (**E**) Changes in blood total cholesterol levels in males with MASLD. Statistical significance between two groups was determined using an unpaired two-tailed Student’s *t*-test. ***, *p* < 0.001; ns, not significant.

This study analyzed data from the NHANES 2013–2016 cohort to examine biomarkers associated with MASLD. From an initial sample of 20,146 participants, a subset of 5244 individuals (2450 males and 2794 females) was selected following exclusion criteria (Figure 1A and Table S3). The analysis demonstrated that male patients with MASLD exhibited significantly lower circulating testosterone levels compared to normal male controls, whereas estradiol levels did not differ significantly (Figure 1B,C). Among males, higher testosterone levels were inversely associated with MASLD, as indicated by HIS (Figure 1D), as well as by FLI and NAFLD-LFS (Tables S4–S6). No similar associations were observed in female participants (Figure S1 and Tables S7–S9). In contrast to the decline in testosterone levels, male MASLD patients showed a significant increase in total blood cholesterol (Figure 1E). Together, these results suggest that decreased circulating testosterone, which inversely correlates with MASLD, represents a distinctive feature of MASLD in males.

### 3.2. Development and Phenotypic Characterization of a Murine MASLD Model

**Figure 2:**
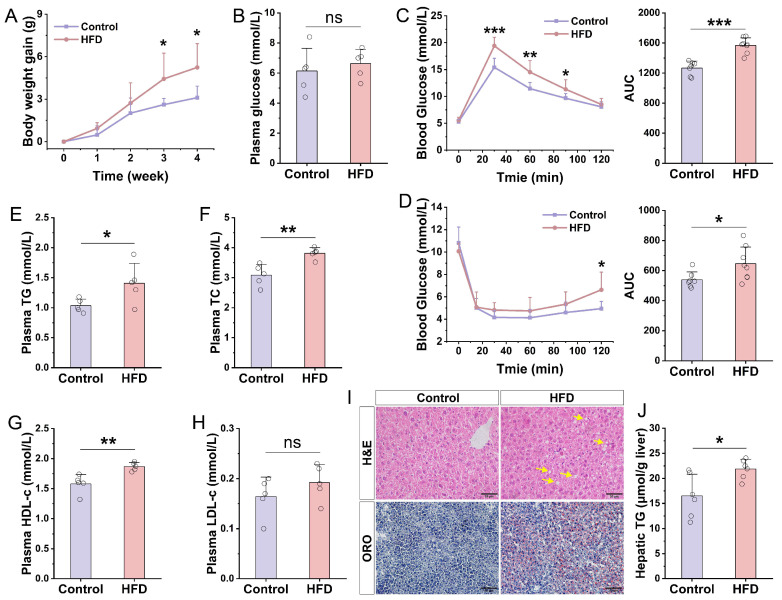
Phenotypic characterization of mice subjected to HFD for a duration of four weeks. (**A**) Changes in body weight (n = 8). (**B**) Fasting blood glucose levels (n = 5). (**C**) Glucose tolerance test (GTT) results alongside area under the curve (AUC) quantification (n = 7–8). (**D**) Insulin tolerance test (ITT) data with corresponding AUC measurements (n = 7–8). (**E**–**H**) Plasma concentrations of triglyceride (TG), total cholesterol (TC), high-density lipoprotein cholesterol (HDL-c), and low-density lipoprotein cholesterol (LDL-c), respectively (n = 5). (**I**) Histological analyses of liver tissue via H&E and Oil Red O (ORO) staining. Yellow arrows indicate lipid droplets in the liver. (**J**) Quantitative assessment of hepatic triglyceride content (n = 6). Statistical significance between two groups was determined using an unpaired two-tailed Student’s *t*-test. *, *p* < 0.05; **, *p* < 0.01; ***, *p* < 0.001; ns, not significant.

The HFD-induced murine model is a well-established system for studying MASLD. To examine the relationship between testosterone and hepatic lipid metabolism in males, adult male C57BL/6 mice were administered an HFD for 4, 8, 16, and 24 weeks to model the temporal progression of MASLD. The 4-week time point was chosen specifically to investigate early disease mechanisms. After 4 weeks of HFD feeding, mice showed a significant increase in body weight (Figure 2A) and developed mild insulin resistance, as indicated by glucose and insulin tolerance test (GTT and ITT), although fasting blood glucose remained unchanged (Figure 2B–D). Plasma levels of TG, TC, and HDL-c were elevated (Figure 2E–G), while LDL-c levels showed no significant alteration (Figure 2H). Histological assessments via H&E and Oil Red O (ORO) staining, together with the quantitative measurement of hepatic TG content, confirmed pronounced lipid accumulation in the liver (Figure 2I,J). Collectively, these findings indicate that 4 weeks of HFD feeding reliably induces early-stage MASLD accompanied by metabolic disturbances, supporting the use of this model for studying initial pathophysiological events in MASLD.

### 3.3. Liver Transcriptomic Analysis Reveals Upregulation of Cholesterol and Steroid Hormone Biosynthesis Pathways in Mouse Livers Following HFD Exposure

**Figure 3:**
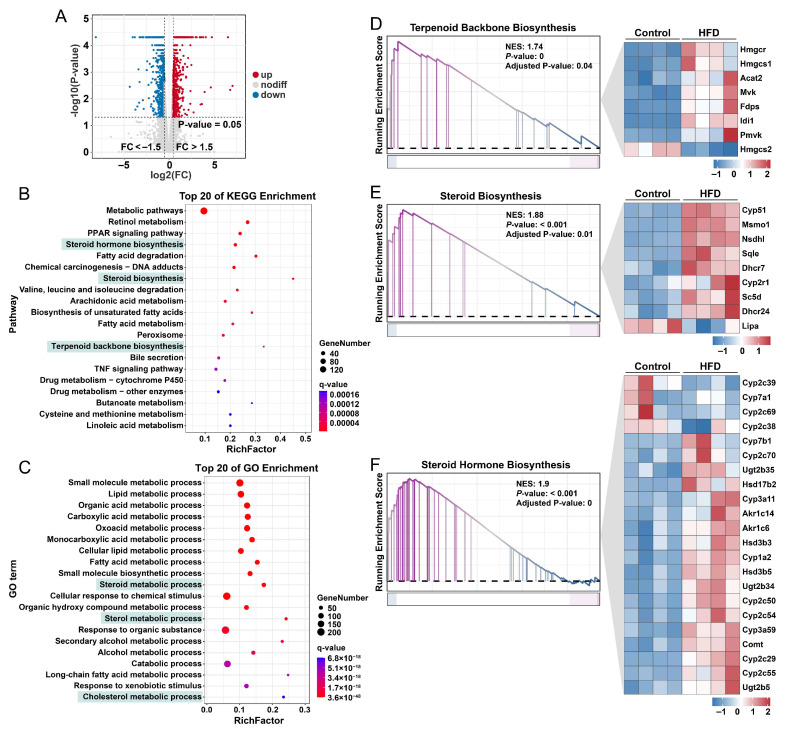
Enhanced expression of hepatic pathways involved in de novo cholesterol biosynthesis and steroid hormone synthesis in mice following four weeks of HFD exposure. (**A**) Differentially expressed genes induced by HFD treatment (fold change > 1.5). (**B**,**C**) The top 20 enriched pathways derived from KEGG and GO analyses, respectively. Pathways highlighted with a green background represent the key biological processes focused on in this study. (**D**–**F**) GSEA results for cholesterol biosynthesis and steroid hormone synthesis pathways, accompanied by heatmaps illustrating expression patterns of relevant genes. Each group comprised four mice designated for liver transcriptome analysis.

To investigate the underlying mechanisms, we performed RNA sequencing on liver tissues from mice after four weeks of HFD feeding. A distinct set of differentially expressed genes was identified (Figure 3A). KEGG pathway analysis revealed significant upregulation of terpenoid backbone biosynthesis, steroid biosynthesis, and steroid hormone biosynthesis pathways in the HFD group (Figure 3B). GO enrichment analysis further supported these findings, showing significant alterations in biological processes related to steroid, sterol, and cholesterol metabolism (Figure 3C). Consistent with these results, gene set enrichment analysis (GSEA) confirmed the heightened activity of steroidogenic pathways following HFD exposure (Figure 3D–F). Together, these data indicate that HFD feeding markedly enhances hepatic steroidogenic and cholesterol biosynthetic pathways in mice.

### 3.4. Liver Metabolomic Analysis Reveals That HFD Induces Significant Alterations in the Hepatic Steroid Hormone Biosynthesis Pathway in Mice

**Figure 4:**
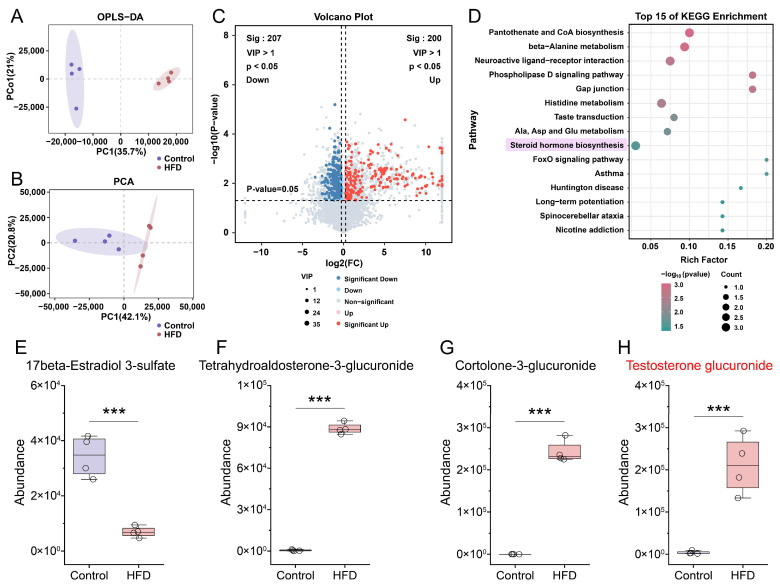
Significant elevation of testosterone metabolite concentrations in the livers of mice after four weeks of HFD administration. (**A**) Orthogonal partial least squares discriminant analysis (OPLS-DA) of hepatic metabolomic profiles. (**B**) Principal component analysis (PCA) of hepatic metabolomic profiles. (**C**) Differential metabolites. (**D**) KEGG pathway enrichment analysis of the differential metabolites. Pathway shaded with a pink background indicates the key biological pathway focused on in this study. (**E**–**H**) Quantification of the abundance of specific steroid hormone metabolites. In panel H, the metabolite name highlighted in red denotes the core metabolite of this study. Each group comprised four mice designated for liver metabolomics analysis. Statistical significance between two groups was determined using an unpaired two-tailed Student’s *t*-test. ***, *p* < 0.001.

Based on the transcriptomic findings, we conducted non-targeted metabolomic profiling of mouse liver samples (Figure 4A,B). A total of 200 metabolites were upregulated, and 207 were downregulated in HFD-fed mice (Figure 4C). KEGG pathway enrichment analysis identified steroid hormone biosynthesis among the top 15 most significantly altered pathways (Figure 4D). Quantitative analyses of steroid hormone metabolites showed decreased levels of 17β-estradiol 3-sulfate (Figure 4E) and elevated levels of tetrahydroaldosterone-3-glucuronide, cortolone-3-glucuronide, and testosterone glucuronide (Figure 4F–H). Given that testosterone glucuronide represents a principal hepatic metabolite of testosterone, these findings collectively suggest that HFD feeding markedly reprograms hepatic steroid hormone metabolism, characterized by an increase in testosterone-derived metabolites.

### 3.5. Integrated Analysis of Hepatic Transcriptomics and Metabolomics and Changes in Liver Cholesterol and Blood Testosterone Levels

**Figure 5:**
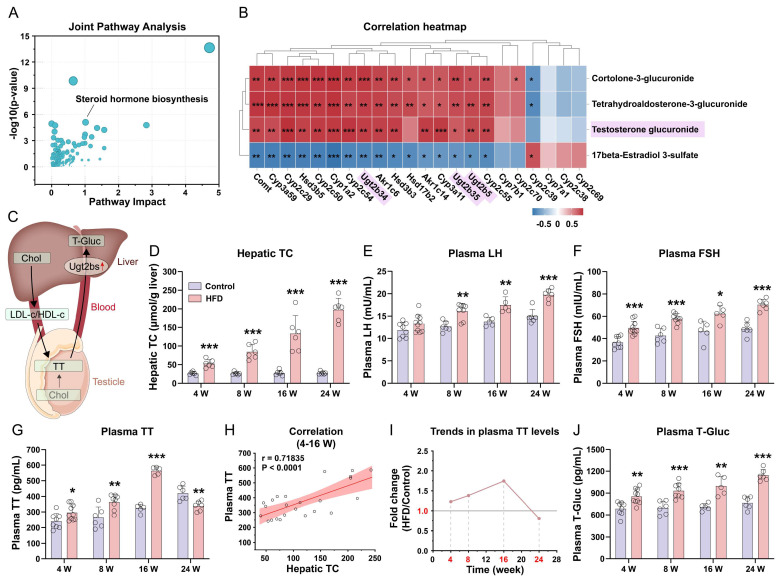
Comprehensive integration of hepatic transcriptomic and metabolomic datasets alongside temporal changes in hepatic cholesterol and circulating testosterone levels. (**A**) Joint pathway analysis of transcriptomic and metabolomic data. (**B**) Correlation heatmap linking steroid hormone-associated genes and metabolites. (**C**) Schematic of cholesterol (Chol), testosterone (TT), and testosterone glucuronide (T-Gluc) flux: TT is synthesized in testicular Leydig cells primarily using liver-derived cholesterol (transported to the testes via LDL-c/HDL-c), alongside Leydig cell-endogenous cholesterol; hepatic Ugt2bs catalyze the conversion of TT to T-Gluc. The red upward arrow next to Ugt2bs highlights the enhanced conversion of TT to T-Gluc identified in this study. (**D**) Hepatic total cholesterol (TC) levels in Control vs. HFD mice at 4, 8, 16, and 24 weeks (n = 6). (**E**) Plasma levels of luteinizing hormone (LH) in Control vs. HFD mice across time points (n = 5–10). (**F**) Plasma levels of follicle-stimulating hormone (FSH) in Control vs. HFD mice across time points (n = 5–10). (**G**) Plasma TT concentrations in Control vs. HFD mice at 4, 8, 16, and 24 weeks (n = 5–10). (**H**) Pearson correlation between hepatic TC and plasma TT (4–16 weeks). (**I**) Temporal fold change (HFD/Control) in plasma TT levels over 24 weeks, showing initial elevation followed by decline. (**J**) Plasma T-Gluc levels in Control vs. HFD mice across time points (n = 5–10). Statistical significance between two groups was determined using an unpaired two-tailed Student’s *t*-test. *, *p* < 0.05; **, *p* < 0.01; ***, *p* < 0.001.

To explore the interactions between differentially expressed genes and metabolites, we performed integrated transcriptomic and metabolomic analysis using MetaboAnalyst. This approach combined enrichment and topological analyses to identify pathways of statistical and biological significance. Steroid hormone biosynthesis emerged as one of the most prominently altered pathways (Figure 5A). The coordinated changes observed at both the transcriptional and metabolic levels indicate a substantial reprogramming of hepatic steroid hormone metabolism in HFD-fed mice, underscoring its potential role in the phenotypic development.

Furthermore, analyses of the human dataset GSE126848 confirmed the upregulation of the steroid hormone biosynthesis pathway in livers from male MASLD patients (Figure S2A), supporting the clinical relevance of our mouse model. Subsequent correlation analysis between steroid hormone-related genes and metabolites revealed a significant positive association between testosterone glucuronide (T-Gluc) and several Ugt2b genes, including Ugt2b5, Ugt2b34, and Ugt2b35 (Figure 5B). As previously established [23], testosterone is synthesized in the testes, a process that critically depends on cholesterol primarily supplied by the liver via circulating lipoproteins and de novo synthesis; moreover, testosterone is inactivated in the liver via Ugt2b-mediated glucuronidation, forming excretable testosterone glucuronide (Figure 5C). Given the elevated T-Gluc levels in HFD mice, we further examined its substrate, testosterone, and testosterone’s precursor, cholesterol. Hepatic TC (Figure 5D) and FC (Figure S3) levels were dramatically elevated in HFD-fed mice across all time points. Concurrently, the plasma levels of gonadotropins (LH and FSH) were increased in HFD mice (Figure 5E,F). Plasma testosterone levels showed a biphasic response: They increased initially (4–16 weeks) and then progressively declined by 24 weeks in HFD-fed mice (Figure 5G). Early-phase testosterone levels (4–16 weeks) correlated positively with hepatic TC accumulation (Figure 5H), and the temporal fold-change in plasma testosterone confirmed this pattern (Figure 5I). In parallel, plasma T-Gluc remained persistently elevated in HFD mice (Figure 5J), indicating enhanced hepatic testosterone clearance. Collectively, these data indicate that HFD-induced hepatic cholesterol synthesis provides substrates for testosterone biosynthesis. During the early stages of MASLD, cholesterol and testosterone levels rise concurrently. As the disease progresses, however, increased UGT2B-mediated glucuronidation likely contributes to the pronounced decline in circulating testosterone.

### 3.6. HFD Induces Hepatic Cholesterol Biosynthesis and Upregulates Testosterone Metabolism Pathways in Mice

**Figure 6:**
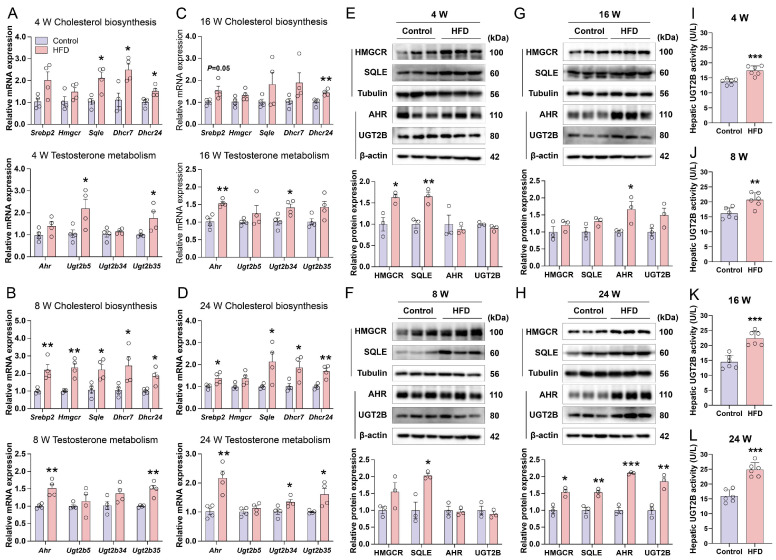
Evaluation of hepatic cholesterol biosynthesis and testosterone metabolism pathways in mice subjected to HFD over varying durations. (**A**–**D**) Gene expression profiles related to hepatic de novo cholesterol synthesis (upper panels) and hepatic testosterone inactivation pathways (lower panels) from 4 to 24 weeks (n = 4). (**E**–**H**) Protein expression analyses and quantitative assessments of both cholesterol biosynthesis and testosterone inactivation pathways across the 4- to 24-week period (n = 3). Note: The observed protein band of UGT2B was detected using a human UGT2B10 antibody, which detected a protein band near 80 kDa. Variations in the intensity of this band correspond with the observed upregulation pattern of Ugt2b34/35 mRNA, suggesting that the detected band likely represents the mouse Ugt2b protein. (**I**–**L**) Hepatic UGT2B enzyme activity (measured as catalytic activity, U/L) in Control vs. HFD mice at 4, 8, 16, and 24 weeks (n = 6). Statistical significance between two groups was determined using an unpaired two-tailed Student’s *t*-test. *, *p* < 0.05; **, *p* < 0.01; ***, *p* < 0.001.

To investigate the mechanisms underlying altered hepatic cholesterol and testosterone metabolism, we analyzed key genes and proteins in these pathways. The mRNA expression of Srebf2-regulated cholesterol biosynthesis genes was consistently elevated at all time points (Figure 6A–D), as were the transcripts of the Ugt2b gene family (Figure 6A–D). Similarly, protein levels of the rate-limiting enzymes HMGCR and SQLE remained persistently elevated throughout HFD feeding (Figure 6E–H).

For UGT2B protein detection, we used a commercially available antibody against human UGT2B10, which cross-reacts with mouse liver lysates. This approach was chosen because of the high sequence homology between human UGT2B10—previously found to be upregulated in the livers of male MASLD patients (Figure S2)—and its mouse orthologs (Ugt2b34, Ugt2b5, and Ugt2b35, see https://www.genecards.org/cgi-bin/carddisp.pl?gene=UGT2B10#orthologs, accessed on 24 November 2025), and it was also chosen due to the lack of commercially validated antibodies specific to the murine Ugt2b isoforms. The protein band detected using this human UGT2B10 antibody was labeled as UGT2B in this study.

Consistent with the transcriptional upregulation of Ugt2b34 and Ugt2b35 at 24 weeks (Figure 6D), a band at ~80 kDa, along with the transcriptional regulator AHR, showed stable intensity at 4 and 8 weeks, a moderate increase at 16 weeks, and a significant rise at 24 weeks of HFD (Figure 6E–H) (note that although the WB result of UGT2B was supported by concordant mRNA expression, we interpret this WB data as indicative rather than definitive evidence). Consistently, we measured hepatic UGT2B enzyme activity across time points. At 4 weeks, HFD mice already exhibited significantly elevated UGT2B activity relative to the controls (Figure 6I). This increase persisted through 8 weeks (Figure 6J) and became further amplified at 16 (Figure 6K) and 24 weeks (Figure 6L). Together, these parallel changes at the mRNA and protein level suggest that in the context of HFD-induced MASLD, hepatic cholesterol biosynthesis may initially support testosterone production. Subsequently, the probable upregulation of UGT2B enzymes promotes testosterone inactivation via glucuronidation, contributing to an overall reduction in circulating testosterone levels as the disease progresses. Nonetheless, the specific functional role of testosterone in this process warrants further investigation.

### 3.7. Exogenous Testosterone Supplementation Attenuates Oleic Acid-Induced Lipid Accumulation by Suppressing Fatty Acid Uptake and Triglyceride Biosynthesis

**Figure 7:**
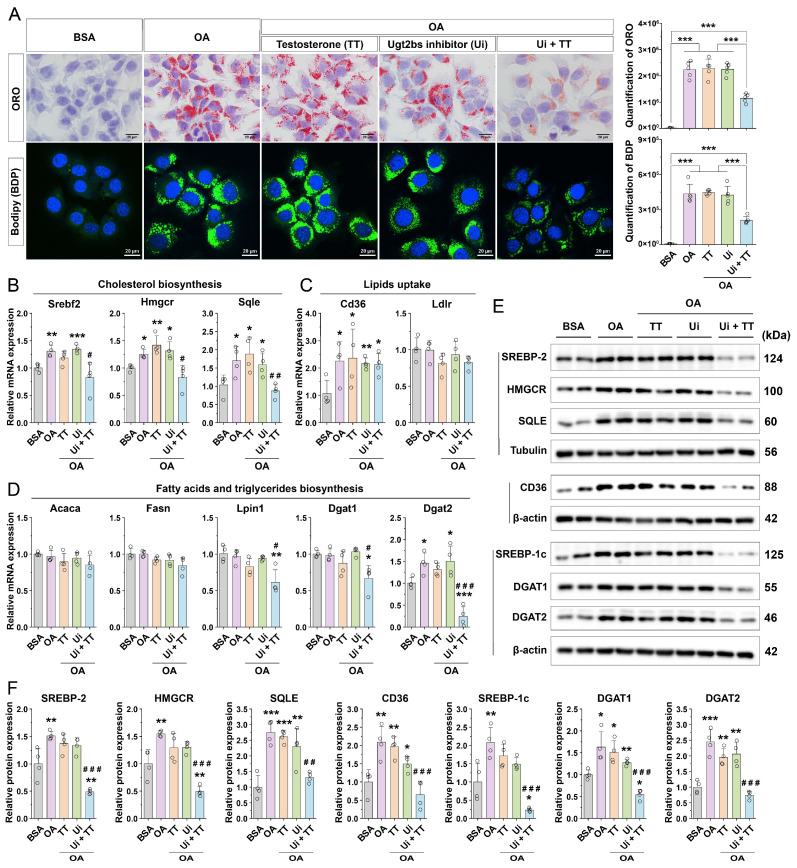
Testosterone mitigates intracellular lipid accumulation in hepatocytes by suppressing fatty acid uptake and triglyceride synthesis. (**A**) ORO and Bodipy (BDP) staining illustrating lipid content in AML-12 cells subjected to various treatment conditions, accompanied by quantitative analysis (n = 5). (**B**) Expression profiles of key genes involved in the cholesterol biosynthesis pathway in AML-12 cells under different treatments (n = 4). (**C**) Expression levels of principal genes associated with lipid uptake pathways in AML-12 cells across treatment groups (n = 4). (**D**) Expression of key genes implicated in de novo fatty acid and triglyceride biosynthesis pathways in AML-12 cells under varying treatment conditions (n = 4). (**E**) Assessment of key proteins related to cholesterol synthesis, fatty acid uptake, and triglyceride synthesis in AML-12 cells following different treatments. (**F**) Quantitative evaluation of Western blot data presented in panel (**E**) (n = 4). Statistical significance among the five groups was determined using one-way ANOVA followed by Tukey’s HSD post hoc test. The pre-specified primary contrast between the OA and Ui+TT groups is highlighted. Comparison between the BSA group and other groups: *, *p* < 0.05; **, *p* < 0.01; ***, *p* < 0.001. Comparison between the OA group and other groups: #, *p* < 0.05; ##, *p* < 0.01; ###, *p* < 0.001.

To examine the role of testosterone in MASLD progression, we established an OA-induced lipid accumulation model in AML-12 hepatocytes. Treatment with 1 μM testosterone (TT) alone did not significantly reduce OA-induced lipid deposition, as shown by Oil Red O (ORO) and BODIPY staining (Figure 7A). Given our earlier in vivo observation of enhanced testosterone inactivation (Figure 6I–L), we hypothesized that OA may activate testosterone-inactivating pathways in hepatocytes, thereby attenuating the effect of testosterone. This was supported by qPCR and WB data (Figure S4).

We next applied the UGT2B inhibitor probenecid (Ui, 30 μM), pretreating cells for 12 h and co-treating with TT for an additional 12 h. The inhibition of testosterone inactivation restored TT’s capacity to reduce lipid accumulation (Figure 7A). Mechanistically, OA stimulation increased mRNA expression of cholesterol biosynthesis genes (Srebf2, Hmgcr, Sqle) (Figure 7B), fatty acid uptake genes (Cd36) (Figure 7C), and triglyceride synthesis genes (Dgat2) (Figure 7D). In the Ui+TT group, the expression of cholesterol biosynthesis genes and Cd36 remained unchanged (Figure 7B,C), while Dgat2 expression was significantly downregulated (Figure 7D). WB analysis confirmed that OA exposure elevated the protein levels of SREBP2, HMGCR, SQLE, CD36, DGAT1, and DGAT2 (Figure 7E,F), and these increases were reversed via Ui+TT co-treatment (Figure 7E,F).

Notably, in the OA-induced AML12 cell model, OA also promoted the upregulation of Ugt2b genes and protein (Figures S4 and S5). Co-treatment with an AHR inhibitor (Ai) and testosterone (TT) significantly reduced lipid deposition (Figure S5). Furthermore, AHR inhibition markedly decreased mRNA levels of Ugt2b5, Ugt2b34, and Ugt2b35 and reduced UGT2B protein expression (Figure S5). Collectively, these results indicate that OA stimulation enhances the cholesterol biosynthesis pathway and upregulates the Ugt2b family gene and protein expression in an AHR-dependent manner. When UGT2B activity is inhibited, testosterone alleviates lipid accumulation by suppressing fatty acid uptake and triglyceride synthesis, thereby attenuating MASLD progression.

## 4. Discussion

This study reveals a novel and dynamic interplay between testosterone and hepatic lipid metabolism in MASLD. In the early phase of HFD feeding, hepatic cholesterol synthesis is upregulated, resulting in increased plasma testosterone levels. As the disease progresses, the AHR-mediated induction of UGT2B expression accelerates testosterone clearance, thereby exacerbating MASLD. Importantly, we show that testosterone possesses intrinsic hepatoprotective properties, but these are obscured by its enhanced metabolic clearance in MASLD. The inhibition of UGT2B restores testosterone’s lipid-lowering activity, highlighting that hepatic metabolism determines its biological action.

Our clinical analysis used NHANES testosterone data from standardized morning samples, minimizing confounding from diurnal variation. This rigorous approach strengthens the reliability of our findings, which confirm prior epidemiological reports of an inverse association between circulating testosterone and MASLD in males [10,11,12,13,14,15]. In HFD-fed mice, we observed a biphasic testosterone trajectory: an initial rise at 4–16 weeks, followed by a pronounced decline at 24 weeks. This temporal pattern—often missed in human cross-sectional studies—suggests a shift from early compensation to later decompensation. The early rise in testosterone coincided with upregulated hepatic cholesterol and steroid biosynthesis, supported by transcriptomic and metabolomic data. This aligns with known hepatic cholesterol overload in MASLD [17,18] and implies that the liver may initially mount a compensatory hormonal response via increased cholesterol synthesis under metabolic stress.

Our results offer a mechanistic explanation for the clinically observed reduction in testosterone levels in men with MASLD [16,37,38]. While the systemic suppression of the HPG axis via adipose tissue-derived factors contributes to this phenomenon [20,21,22], our data indicate that testosterone deficiency in advanced MASLD stems primarily from enhanced hepatic clearance. Integrated multi-omics and experimental validation consistently indicated the activation of testosterone glucuronidation in HFD-fed mice. This was evidenced by the following: upregulation of Ugt2b family genes (Ugt2b5, Ugt2b34, and Ugt2b35), elevated hepatic testosterone glucuronide, increased UGT2B protein levels, enhanced hepatic UGT2B activity, and induction of its transcriptional regulator AHR. In the cellular model, AHR inhibition significantly decreased Ugt2bs mRNA and UGT2B protein levels, indicating that OA-induced Ugt2bs upregulation is largely AHR-dependent. These findings align with the established role of AHR in regulating UGT2B enzymes [26,27] and in HFD-induced metabolic disturbances [28,29]. Furthermore, analyses of human transcriptomic data (GSE126848) confirmed the upregulation of steroid hormone biosynthesis pathways in the livers of male MASLD patients, supporting the translational relevance of this mechanism.

The critical role of testosterone bioavailability in hepatic lipid metabolism was clearly demonstrated in vitro. Exogenous testosterone alone did not reduce OA-induced lipid accumulation in AML-12 hepatocytes, underscoring the importance of hepatic clearance. However, pharmacological inhibition of UGT2B with probenecid [39,40,41] restored testosterone’s ability to attenuate lipid deposition. Mechanistically, testosterone alleviated MASLD through a dual mechanism: (1) suppressing fatty acid uptake via the post-translational downregulation of the CD36 protein—potentially through ubiquitin-mediated degradation [42]—without changing its mRNA level; and (2) inhibiting triglyceride synthesis by downregulating DGAT1 and DGAT2 at both transcriptional and protein levels. These findings establish a direct hepatoprotective role for testosterone in MASLD, mediated through both the post-translational and transcriptional regulation of lipid metabolic pathways.

From a therapeutic perspective, testosterone supplementation in MASLD has exhibited inconsistent outcomes. While some studies report improvement [43,44,45], others find no significant benefit [46,47] and note potential risks, such as increased prostate cancer incidence [48]. Our data suggest that systemic testosterone administration may be ineffective in advanced MASLD, likely due to accelerated hepatic clearance. Thus, hepatic UGT2B activity emerges as a potential therapeutic target. However, systemic UGT inhibition (e.g., with probenecid) is not clinically feasible because it broadly affects the metabolism of numerous endogenous and xenobiotic compounds. Future translational efforts should therefore focus on developing liver-selective UGT2B modulators that can locally enhance testosterone signaling while minimizing systemic exposure. Achieving this will require strategies such as liver-directed prodrugs or targeted conjugates, along with the careful preclinical evaluation of potential off-target effects on drug metabolism and endocrine homeostasis.

Our proposed mechanism differs from established models of endocrine dysfunction in MASLD. While the “adipopathic” model emphasizes systemic HPG axis suppression via pro-inflammatory adipokines [49], our data highlight a critical hepatic determinant. We show that in advanced MASLD, the HPG axis remains compensatory—evident from elevated LH and FSH—yet this response is offset by accelerated hepatic testosterone clearance via AHR-mediated UGT2B upregulation. This “metabolic clearance” hypothesis explains the clinical paradox of low testosterone levels coexisting with active MASLD: Testosterone is not merely underproduced but is actively cleared by the diseased liver. While the AHR is well characterized as a xenobiotic sensor, it is increasingly recognized to play a role in metabolic adaptation during obesity [50]. Moving beyond this established metabolic role, our study identifies a novel function for AHR in the clearance of an endogenous hormone. Specifically, we demonstrate that AHR activation upregulates hepatic UGT2B expression, thereby accelerating testosterone glucuronidation. This finding suggests a potential therapeutic strategy aimed at the AHR-UGT2B axis. This approach, particularly through modulating hepatic UGT2B activity, seeks to restore testosterone bioavailability and thereby alleviate MASLD.

This study provides a detailed analysis of dynamic changes in testosterone metabolism during MASLD progression, supported by multi-level evidence. Key strengths include integrative multi-omics, longitudinal assessment across disease stages, and functional rescue experiments. The findings in mice are consistent with human transcriptomic data (GSE126848 and GSE130970), supporting the translational relevance of the proposed mechanism. However, several limitations should be noted. The reliance on a single dietary model necessitates validation in alternative models (e.g., MCD diet or genetic models). The use of the pharmacological inhibitor probenecid, while supportive, constitutes a limitation. Probenecid is a broad-spectrum inhibitor of organic anion transporters and multiple UGT enzymes; therefore, its rescuing effect on testosterone activity, although consistent with our hypothesis, cannot definitively establish a UGT2B-specific mechanism due to potential off-target effects. Genetic interventions—such as siRNA/CRISPR-mediated knockdown of specific Ugt2b isoforms in cells and hepatocyte-specific Ugt2b isoform knockout mice—would be required to provide more conclusive causal evidence and are an important direction for future research. The precise role of AHR in regulating UGT2B-mediated testosterone clearance in HFD and OA models also requires further investigation. Furthermore, due to the lack of isoform-specific protein validation tools for mouse Ugt2b isoforms, the protein evidence for UGT2B induction relies on a human UGT2B10 antibody that cross-reacts with mouse lysates. The observed ~80 kDa UGT2B band may represent one or more mouse Ugt2b isoforms. While this provides indicative support, the direct link between the observed changes in testosterone glucuronidation and a specific Ugt2b isoform remains inferential and requires future confirmation with isoform-specific tools (e.g., isoform-specific antibodies or mass spectrometry). Finally, while the concurrent elevation of hepatic testosterone glucuronide, UGT2B activity, and plasma LH/FSH supports a model where accelerated hepatic clearance is a key contributor to low testosterone in advanced MASLD, our study does not fully resolve the quantitative balance between this mechanism and potential alterations in the HPG axis. We interpret the elevated LH/FSH as a compensatory response to the low testosterone state; however, we cannot definitively exclude alternative explanations, such as changes in gonadotropin clearance itself. Future studies employing tracer kinetics or direct assessments of testicular steroidogenic capacity are needed to precisely determine the relative contributions of hepatic metabolism and central endocrine pathways to testosterone deficiency in progressive MASLD.

## 5. Conclusions

In summary, this study indicates that a high-fat diet promotes MASLD progression by upregulating CD36-mediated fatty acid uptake and enhancing triglyceride synthesis through DGAT1 and DGAT2. Although fatty acid-induced cholesterol biosynthesis initially elevates testosterone production, which can counteract lipid accumulation, the concurrent activation of the AHR-UGT2B pathway accelerates testosterone clearance and diminishes its protective effects. These results delineate a regulatory circuit that links fatty acid exposure, cholesterol synthesis, AHR-UGT2B signaling, testosterone clearance, and the resulting metabolic phenotype. Furthermore, they extend our understanding of hypotestosteronemia associated with MASLD by highlighting the role of peripheral hepatic metabolic clearance, thereby complementing the classical view of central HPG axis suppression.

## Data Availability

Publicly available datasets are located at: https://wwwn.cdc.gov/nchs/nhanes/ (accessed on 24 November 2025). The original contributions presented in the study are included in the article/Supplementary Materials; further inquiries can be directed to the corresponding authors.

## Supplementary Materials

The following supporting information can be downloaded at: https://www.mdpi.com/article/10.3390/nu18030549/s1, Table S1. List of primers used in this study; Table S2. List of antibodies used in this study; Table S3. Descriptive analysis of demographic characteristics, anthropometric measures, and blood biochemical parameters of human individuals: data from the National Health and Nutrition Examination Survey (NHANES), 2013–2016; Table S4. Multivariable odds ratios of risk factors for MASLD using Hepatic Steatosis Index (HIS) according to SHBG levels in men; Table S5. Multivariable odds ratios of risk factors for MASLD using Fatty Liver Index (FLI) according to sex hormone levels in men; Table S6. Multivariable odds ratios of risk factors for MASLD using Non-Alcoholic Fatty Liver Disease-Liver Fat Score (NAFLD-LFS) according to sex hormone levels in men; Table S7. Multivariable odds ratios of risk factors for MASLD using Hepatic Steatosis Index (HIS) according to sex hormone levels in women; Table S8. Multivariable odds ratios of risk factors for MASLD using Fatty Liver Index (FLI) according to sex hormone levels in women; Table S9. Multivariable odds ratios of risk factors for MASLD using Non-Alcoholic Fatty Liver Disease-Liver Fat Score (NAFLD-LFS) according to sex hormone levels in women; Figure S1. Evaluation of pertinent biomarkers in the female population derived from NHANES data; Figure S2. Analysis of hepatic transcriptomic data from male patients with MASLD; Figure S3. Temporal assessment of hepatic free cholesterol concentrations in mice subjected to HFD; Figure S4. Impact of oleic acid (OA) and testosterone treatments on the testosterone inactivation pathway within an OA-induced cellular model; Figure S5. AHR is required for OA-induced UGT2B upregulation and consequent testosterone inactivation in AML-12 cells.
