# Supplementary material for: Hepatic UGT2B-Mediated Testosterone Clearance Promotes Lipid Accumulation in High-Fat-Diet-Induced MASLD
Source: Nutrients. 2026 Feb 6;18(3):549. doi: 10.3390/nu18030549 (PMC12899675; doi:10.3390/nu18030549)
Supplement: Supplementary file 1 [file nutrients-18-00549-s001.zip › nutrients-4033509-supplementary.pdf]

Supplementary Materials

Supplementary Materials and Methods

Cell Culture and treatment with AHR inhibitor

The AML-12 mouse hepatocyte cell line was maintained in Dulbecco's Modified Eagle Medium (C11995500CP, Gibco) supplemented with 10% fetal bovine serum (10099-141; Gibco) and 100 U/mL penicillin-streptomycin. Cultures were incubated at 37°C in a humidified atmosphere containing 5% CO2.

To induce lipid accumulation, oleic acid (OA) (O3880; Sigma-Aldrich) was com-plexed with 10% (w/v) fatty acid-free bovine serum albumin (BSA) in PBS under gentle heating (37 °C) and vortexing. Cells were then exposed to this OA-BSA complex at a final OA concentration of 0.2 mM for 24 hours. To assess the potential effect of AHR on Utg2bs expression, cells were first treated with 40 µM of CH-223191 (T2448, TargetMol; a widely used AHR inhibitor, termed Ai) together with OA for 12 hours. Then, 1 µM testosterone (Zhiyi Biotech) was added, and the cells were incubated for another 12 hours in the continued presence of OA and Ai.

Supplementary Tables

Primer symbols	Primer sequences (5'-3')	
	Forward	Reverse
Srebf2	CCAAGGAGAGCCTGTACTGC	CCTGAGGTTTCACCAAGGAC
Hmgcr	CCGAATTGTATGTGGCACTG	GGTGCACGTTCCTTGAAGAT
Sqle	CAGCTTCCTTCCTCCTTCCT	TCAGTCTGGGCCGTTAAGAC
Dhcr7	CATCGGGAAGTGGTTTGACT	CAGCCTAGGTACCACCCAAA
Dhcr24	CCCTGGTTCTTCAAGCATGT	CGAAGAGGTAGCGGAAGATG
Cd36	TGCTTGCAAATCCAAGAATG	AGAGAGAGCACACACCACCA
Ldlr	GCCACATGGTATGGGTTCC	GCTCGTCCTCTGTGGTCTTC
Acaca	ATGTTCCCAGCCTGTAATGG	AGCCAAGCGGATGTAAACTG
Fasn	TGCACCTCACAGGCATCAAT	GTCCCACTTGATGTGAGGGG
Lpin1	CATGCTTCGGAAAGTCCTTCA	GGTTATTCTTTGGCGTCAACCT
Dgat1	GCTGATCCCAGGTTGTTCAT	GAGACAGCTTTGGCCTTGAC
Dgat2	ACGCAGTCACCCTGAAGAAC	CCCAGGTGTCAGAGGAGAAG
Ahr	CTGGTTGTCACAGCAGATGCCT	CGGTCTTCTGTATGGATGAGCTC
Ugt2b35	CCTGCTAAGCCCTTGCCTAAG	AAATTGCGTTGGCCCTTTCTT
Ugt2b34	GGCTCCAACACTCGTCTGTA	TAACAGCTGCTCCTTTGGCC
Ugt2b5	TATGGTGGCCAAAGGAGCAG	GGTGCTTGGCTCTTTTGTGG
β-Actin	GGCTGTATTCCCCTCCATCG	CCAGTTGGTAACAATGCCATGT

Table S1. List of primers used in this study.

Antibody	Supplier	Product code
SREBP-1c	Affinity	AF6283
DGAT1	Abiowell	AWA12792
DGAT2	Abiowell	AWA54021
CD36	Abcam	Ab133625
SREBP2	Proteintech	28212-1-AP
HMGCR	ABclonal	A19063
SQLE	ABclonal	A2428
AHR	Proteintech	67785-1-Ig
UGT2B10	ABclonal	A7570
Tubulin	CST	2125S
β-Actin	CST	4967L

Table S2. List of antibodies used in this study.

	**Male**		**Female**	
	**Normal** (n=1284)	**MASLD** (n=1166)	**Normal** (n=1609)	**MASLD** (n=1185)
Age (years)	34.45±12.76	40.47±11.56	35.37±12.12	41.43±11.31
BW (kg)	74.06±11.33	102.28±20.97***	64.33±11.59	95.49±20.31***
Height (cm)	174.25±7.57	174.88±7.85	161.06±6.89	160.98±7.03
BMI	24.34±3.14	33.33±5.85***	24.77±3.99	36.71±6.77***
WC (cm)	87.15±9.09	111.53±13.90***	85.41±9.63	113.26±14.12***
TG (mmol/L)	0.98±0.53	2.04±1.81***	0.87±0.49	1.59±2.26***
TC (mmol/L)	4.56±0.96	5.12±1.12***	4.67±0.95	5.03±1.04***
HDL-c (mmol/L)	1.33±0.33	1.06±0.26***	1.57±0.40	1.26±0.33***
LDL-c (mmol/L)	2.78±0.87	3.18±0.93***	2.71±0.87	3.02±0.84***
FBG (mmol/L)	5.62±1.31	6.49±2.50***	5.30±1.08	6.43±2.53***
AST (U/L)	25.78±15.84	30.08±29.16***	21.44±7.89	25.66±29.07***
ALT (U/L)	24.76±15.83	38.01±28.36***	18.44±11.36	26.17±20.35***
GGT (U/L)	20.13±13.97	39.48±47.90***	15.56±11.23	33.01±49.88***

Table S3. Descriptive analysis of demographic characteristics, anthropometric measures, and blood biochemical parameters of human individuals: data from the National Health and Nutrition

Examination Survey (NHANES), 2013-2016. Data are presented as mean±SD. BW: body weight; BMI: body mass index; WC: waist circumference; TG: triglycerides; TC: total cholesterol; HDL-c: high-density lipoprotein cholesterol; LDL-c: low-density lipoprotein cholesterol; FBG: fasting blood glucose; AST: aspartate aminotransferase; ALT: alanine aminotransferase; GGT: gamma-glutamyl transferase. Statistical significance was determined by unpaired two-tailed Student's t-test. ***: $P < 0.001$, which reflects the statistical significance between MASLD and Normal within the same gender group.

	**Multivariable model 1**		**Multivariable model 2**		**Multivariable model 3**	
	**OR (95% CI)**	***P* value**	**OR (95% CI)**	***P* value**	**OR (95% CI)**	***P* value**
SHBG (nmol/L)						
Quartile 1	1		1		1	
Quartile 2	0.47 (0.27-0.80)	0.006	0.55 (0.29-1.01)	0.053	0.35 (0.13-0.87)	0.026
Quartile 3	0.20 (0.11-0.35)	<0.001	0.36 (0.19-0.68)	0.002	0.41 (0.15-1.05)	0.065
Quartile 4	0.08 (0.04-0.14)	<0.001	0.13 (0.06-0.27)	<0.001	0.14 (0.04-0.40)	<0.001

Table S4. Multivariable odds ratios of risk factors for MASLD using Hepatic Steatosis Index (HIS) according to SHBG levels in men. Multivariable model 1 was adjusted for age and alcohol consumption; Multivariable model 2 was adjusted for age, alcohol consumption, insulin resistance (HOMA-IR), smoking status, and medication use; Multivariable model 3 includes BMI in addition to the variables addressed in model 2. SHBG: Quartile 1, < 23.77 nmol/L; Quartile 2, 23.77 - 32.22 nmol/L; Quartile 3, 32.22 - 45.09 nmol/L; Quartile 4, ≥45.09 nmol/L.

	Multivariable model 1		Multivariable model 2		Multivariable model 3	
	**OR (95% CI)**	***P* value**	**OR (95% CI)**	***P* value**	**OR (95% CI)**	***P* value**
Testasterone (ng/dL)						
Quartile 1	1		1		1	
Quartile 2	0.47 (0.28-0.77)	0.003	0.86 (0.47-1.56)	0.615	1.07 (0.48-2.42)	0.861
Quartile 3	0.22 (0.13-0.36)	<0.001	0.50 (0.27-0.93)	0.030	0.74 (0.32-1.69)	0.474
Quartile 4	0.13 (0.07-0.22)	<0.001	0.29 (0.14-0.56)	<0.001	0.81 (0.31-2.10)	0.661
SHBG (nmol/L)						
Quartile 1	1		1		1	
Quartile 2	0.55 (0.33-0.92)	0.024	0.61 (0.33-1.12)	0.116	0.44 (0.19-1.01)	0.056
Quartile 3	0.18(0.10-0.32)	<0.001	0.27 (0.14-0.52)	<0.001	0.22 (0.08-0.54)	0.001
Quartile 4	0.13 (0.07-0.23)	<0.001	0.21 (0.10-0.42)	<0.001	0.26 (0.09-0.71)	0.009

Table S5. Multivariable odds ratios of risk factors for MASLD using Fatty Liver Index (FLI) according to sex hormone levels in men. Multivariable model 1 was adjusted for age and alcohol consumption; Multivariable model 2 was adjusted for age, alcohol consumption, insulin resistance (HOMA-IR), smoking status, and medication use; Multivariable model 3 includes BMI in addition to the variables addressed in model 2. Testasterone: Quartile 1, < 348.00 ng/dL; Quartile 2, 348.00-452.50 ng/dL; Quartile 3, 452.50-556.47 ng/dL; Quartile 4, ≥556.47 ng/dL. SHBG: Quartile 1, < 23.77 nmol/L; Quartile 2, 23.77-32.22 nmol/L; Quartile 3, 32.22-45.09 nmol/L; Quartile 4, ≥45.09 nmol/L.

	Multivariable model 1		Multivariable model 2		Multivariable model 3	
	**OR (95% CI)**	***P* value**	**OR (95% CI)**	***P* value**	**OR (95% CI)**	***P* value**
Testasterone (ng/dL)						
Quartile 1	1		1		1	
Quartile 2	0.32 (0.20-0.53)	<0.001	0.60 (0.28-1.30)	0.195	0.62 (0.28-1.36)	0.233
Quartile 3	0.14 (0.08-0.24)	<0.001	0.40 (0.17-0.89)	0.027	0.44 (0.19-1.03)	0.060
Quartile 4	0.11 (0.06-0.19)	<0.001	0.29 (0.12-0.72)	0.008	0.42 (0.16-1.06)	0.067
SHBG (nmol/L)						
Quartile 1	1		1		1	
Quartile 2	0.43 (0.26-0.71)	0.001	0.33 (0.14-0.73)	0.007	0.31 (0.13-0.71)	0.006
Quartile 3	0.15 (0.09-0.27)	<0.001	0.21 (0.08-0.50)	<0.001	0.20 (0.07-0.49)	<0.001
Quartile 4	0.16 (0.09-0.28)	<0.001	0.39 (0.15-0.95)	0.040	0.48 (0.18-1.20)	0.120

Table S6. Multivariable odds ratios of risk factors for MASLD using Non-Alcoholic Fatty Liver Disease-Liver Fat Score (NAFLD-LFS) according to sex hormone levels in men. Multivariable model 1 was adjusted for age and alcohol consumption; Multivariable model 2 was adjusted for age, alcohol consumption, insulin resistance (HOMA-IR), smoking status, and medication use; Multivariable model 3 includes BMI in addition to the variables addressed in model 2. Testasterone: Quartile 1, < 348.00 ng/dL; Quartile 2, 348.00-452.50 ng/dL; Quartile 3, 452.50-556.47 ng/dL; Quartile 4, ≥556.47 ng/dL. SHBG: Quartile 1, < 23.77 nmol/L; Quartile 2, 23.77-32.22 nmol/L; Quartile 3, 32.22-45.09 nmol/L; Quartile 4, ≥45.09 nmol/L.

	Multivariable model 1		Multivariable model 2		Multivariable model 3	
	OR (95% CI)	*P* value	OR (95% CI)	*P* value	OR (95% CI)	*P* value
Testosterone (ng/dL)						
Quartile 1	1		1		1	
Quartile 2	1.02 (0.64-1.65)	0.920	1.15 (0.65-2.02)	0.631	0.34 (0.11-0.99)	0.052
Quartile 3	1.03 (0.64-1.68)	0.889	1.22 (0.69-2.17)	0.486	0.32 (0.10-1.01)	0.056
Quartile 4	0.97 (0.59-1.59)	0.902	1.31 (0.73-2.37)	0.366	0.28 (0.08-0.92)	0.041
SHBG (nmol/L)						
Quartile 1	1		1		1	
Quartile 2	0.47 (0.28-0.79)	0.005	0.86 (0.46-1.59)	0.632	0.95 (0.29-3.13)	0.934
Quartile 3	0.21 (0.12-0.34)	<0.001	0.52 (0.28-0.94)	0.032	0.49 (0.15-1.60)	0.238
Quartile 4	0.18 (0.11-0.30)	<0.001	0.40 (0.21-0.74)	0.003	0.30 (0.08-1.08)	0.069
FT (nmol/L)						
Quartile 1	1		1		1	
Quartile 2	1.74 (1.09-2.78)	0.021	1.59 (0.92-2.77)	0.100	0.42 (0.14-1.25)	0.125
Quartile 3	1.87 (1.17-3.02)	0.010	1.75 (1.00-3.07)	0.050	0.65 (0.21-1.97)	0.450
Quartile 4	7.02 (4.13-12.20)	<0.001	4.50 (2.40-8.62)	<0.001	1.60 (0.44-5.89)	0.475

Table S7. Multivariable odds ratios of risk factors for MASLD using Hepatic Steatosis Index (HIS) according to sex hormone levels in women. Multivariable model 1 was adjusted for age and alcohol consumption; Multivariable model 2 was adjusted for age, alcohol consumption, insulin resistance (HOMA-IR), smoking status, and medication use; Multivariable model 3 includes BMI in addition to the variables addressed in model 2. Testosterone: Quartile 1, < 15.90 ng/dL; Quartile 2, 15.90-22.10 ng/dL; Quartile 3, 22.10-31.60 ng/dL; Quartile 4, ≥ 31.60 ng/dL. SHBG: Quartile 1, < 37.70 nmol/L; Quartile 2, 37.70-54.87 nmol/L; Quartile 3, 54.87-86.63 nmol/L; Quartile 4, ≥86.63 nmol/L. Free Testasterone (FT): Quartile 1, < 0.01 nmol/L; Quartile 2, 0.01-0.02 nmol/L; Quartile 3, 0.02-0.04 nmol/L; Quartile 4, ≥0.04 nmol/L.

	Multivariable model 1		Multivariable model 2		Multivariable model 3	
	**OR (95% CI)**	***P* value**	**OR (95% CI)**	***P* value**	**OR (95% CI)**	***P* value**
Testasterone (ng/dL)						
Quartile 1	1		1		1	
Quartile 2	1.00 (0.63-1.59)	0.995	1.12 (0.62-2.02)	0.705	0.48 (0.20-1.14)	0.101
Quartile 3	1.10 (0.68-1.77)	0.703	1.49 (0.83-2.70)	0.186	0.61 (0.24-1.54)	0.298
Quartile 4	1.02 (0.62-1.68)	0.946	1.52 (0.82-2.85)	0.184	0.43 (0.16-1.12)	0.086
SHBG (nmol/L)						
Quartile 1	1		1		1	
Quartile 2	0.43 (0.27-0.68)	<0.001	0.74 (0.41-1.32)	0.307	0.78 (0.34-1.79)	0.558
Quartile 3	0.22 (0.13-0.36)	<0.001	0.59 (0.33-1.07)	0.085	0.56 (0.22-1.40)	0.220
Quartile 4	0.24 (0.14-0.39)	<0.001	0.50 (0.27-0.93)	0.028	0.53 (0.21-1.35)	0.186
FT (nmol/L)						
Quartile 1	1		1		1	
Quartile 2	2.13 (1.29-3.53)	0.003	2.29 (1.26-4.22)	0.007	1.57 (0.62-4.02)	0.338
Quartile 3	1.99 (1.20-3.34)	0.008	2.23 (1.21-4.15)	0.011	1.56 (0.60-4.12)	0.367
Quartile 4	5.77 (3.43-9.90)	<0.001	3.69 (1.96-7.07)	<0.001	1.43 (0.54-3.81)	0.473

Table S8. Multivariable odds ratios of risk factors for MASLD using Fatty Liver Index (FLI) according to sex hormone levels in women. Multivariable model 1 was adjusted for age and alcohol consumption; Multivariable model 2 was adjusted for age, alcohol consumption, insulin resistance (HOMA-IR), smoking status, and medication use; Multivariable model 3 includes BMI in addition to the variables addressed in model 2. Testasterone: Quartile 1, < 15.90 ng/dL; Quartile 2, 15.90-22.10 ng/dL; Quartile 3, 22.10-31.60 ng/dL; Quartile 4, ⩾31.60 ng/dL. SHBG: Quartile 1, < 37.70 nmol/L; Quartile 2, 37.70-54.87 nmol/L; Quartile 3, 54.87-86.63 nmol/L; Quartile 4, ⩾86.63 nmol/L. Free Testasterone (FT): Quartile 1, < 0.01 nmol/L; Quartile 2, 0.01-0.02 nmol/L; Quartile 3, 0.02-0.04 nmol/L; Quartile 4, ⩾0.04 nmol/L.

	Multivariable model 1		Multivariable model 2		Multivariable model 3	
	**OR (95% CI)**	***P* value**	**OR (95% CI)**	***P* value**	**OR (95% CI)**	***P* value**
Testasterone (ng/dL)						
Quartile 1	1		1		1	
Quartile 2	0.80 (0.50-1.28)	0.352	0.68 (0.28-1.62)	0.380	0.63 (0.26-1.52)	0.306
Quartile 3	0.96 (0.59-1.54)	0.854	1.56 (0.66-3.70)	0.310	1.34 (0.56-3.23)	0.515
Quartile 4	0.59 (0.35-0.99)	0.046	0.66 (0.25-1.71)	0.397	0.61 (0.23-1.57)	0.305
SHBG (nmol/L)						
Quartile 1	1		1		1	
Quartile 2	0.41 (0.26-0.65)	<0.001	1.05 (0.47-2.36)	0.908	1.04 (0.46-2.34)	0.927
Quartile 3	0.15 (0.09-0.25)	<0.001	0.70 (0.30-1.65)	0.420	0.72 (0.30-1.69)	0.450
Quartile 4	0.17 (0.10-0.28)	<0.001	0.52 (0.21-1.27)	0.157	0.56 (0.22-1.38)	0.212
FT (nmol/L)						
Quartile 1	1		1		1	
Quartile 2	1.75 (1.05-2.93)	0.033	1.89 (0.77-4.73)	0.165	1.67 (0.67-4.23)	0.273
Quartile 3	1.59 (0.95-2.70)	0.081	1.45 (0.58-3.64)	0.425	1.29 (0.51-3.28)	0.594
Quartile 4	5.12 (3.05-8.77)	<0.001	2.34 (0.92-6.07)	0.076	1.95 (0.74-5.19)	0.177

Table S9. Multivariable odds ratios of risk factors for MASLD using Non-Alcoholic Fatty Liver Disease-Liver Fat Score (NAFLD-LFS) according to sex hormone levels in women. Multivariable model 1 was adjusted for age and alcohol consumption; Multivariable model 2 was adjusted for age, alcohol consumption, insulin resistance (HOMA-IR), smoking status, and medication use; Multivariable model 3 includes BMI in addition to the variables addressed in model 2. Testasterone: Quartile 1, < 15.90 ng/dL; Quartile 2, 15.90-22.10 ng/dL; Quartile 3, 22.10-31.60 ng/dL; Quartile 4, ⩾ 31.60 ng/dL. SHBG: Quartile 1, < 37.70 nmol/L; Quartile 2, 37.70-54.87 nmol/L; Quartile 3, 54.87-86.63 nmol/L; Quartile 4, ⩾86.63 nmol/L. Free Testasterone (FT): Quartile 1, < 0.01 nmol/L; Quartile 2, 0.01-0.02 nmol/L; Quartile 3, 0.02-0.04 nmol/L; Quartile 4, ⩾0.04 nmol/L.

Supplementary Figures

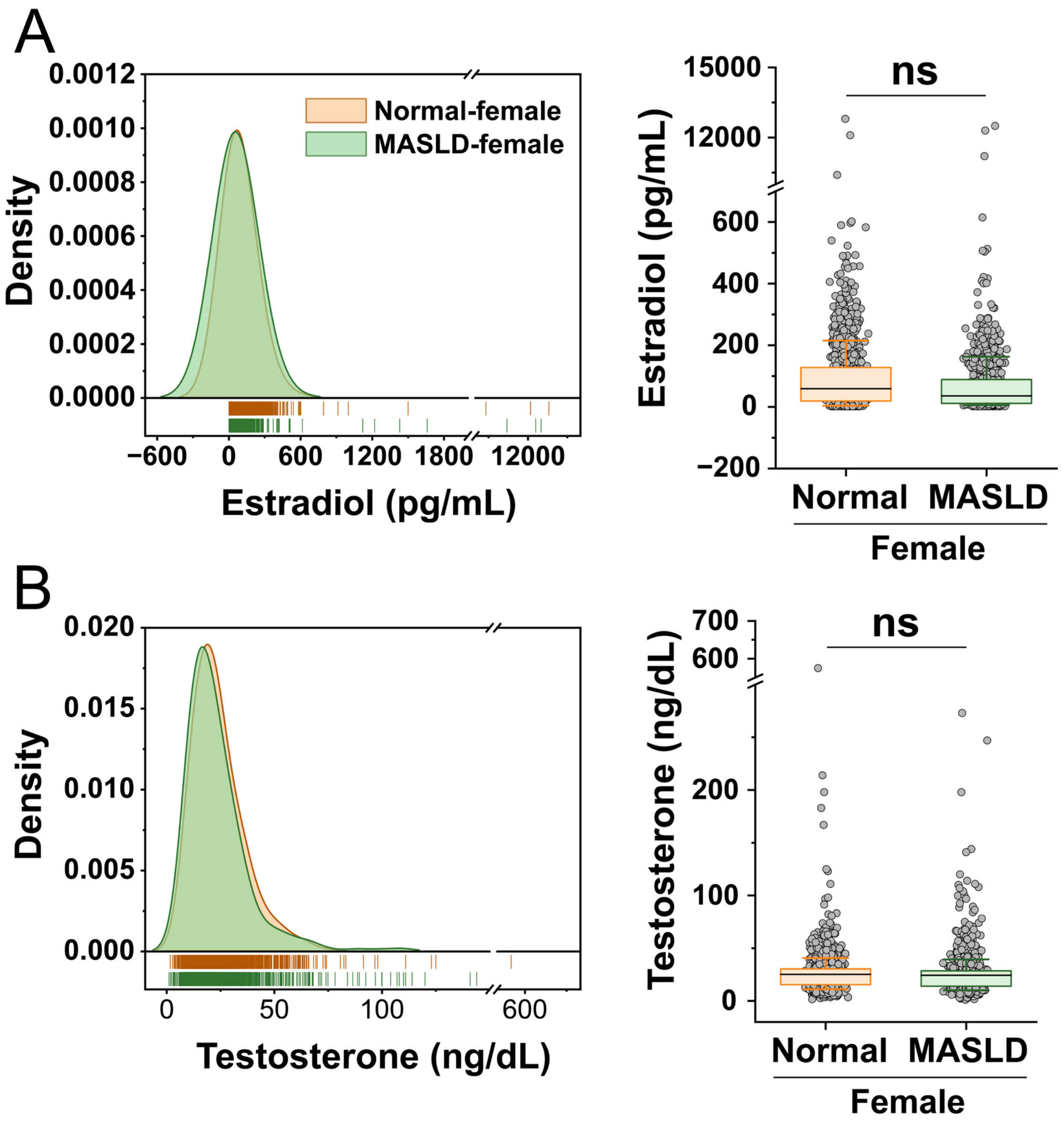

Figure S1. Evaluation of pertinent biomarkers in the female population derived from NHANES data. (A) Alterations in circulating estradiol levels among female patients with MASLD. (B) Changes in blood testosterone levels in female MASLD patients. Data are presented as mean ± SD. Statistical significance was determined by unpaired two-tailed Student's t-test. ns, not significant.

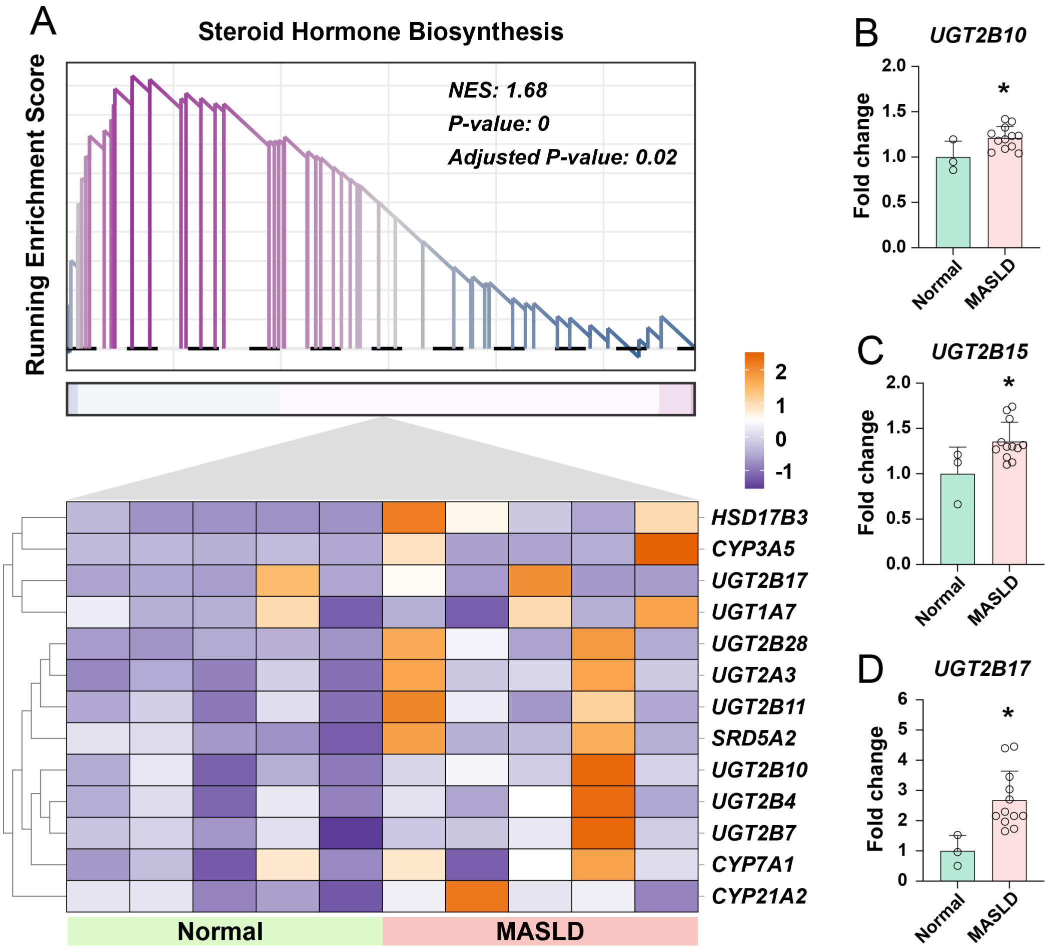

Figure S2. Analysis of hepatic transcriptomic data from male patients with MASLD. (A) Gene set enrichment analysis (GSEA) of the GSE126848 dataset revealed a significant upregulation of the steroid hormone biosynthesis pathway in the livers of male patients with MASLD (n=5). (B–D) Fold change in expression of UGT2B10 (B), UGT2B15 (C), and UGT2B17 (D) in liver transcriptome data (GSE130970) from male patients with MASLD (n=3-12). Data are presented as mean ± SD. Statistical significance was determined by unpaired two-tailed Student's t-test. *, P<0.05.

Note on data exclusion and analysis: Analyses for both datasets were performed exclusively on data from male patients; female patient data were excluded. For gene set enrichment analysis (GSEA) in panel A, gene expression matrix from GSE126848 were analyzed using OECloud tools (https://cloud.oebiotech.com) with default parameters to calculate enrichment scores, nominal p-values, and false discovery rates (FDR), as described in the Materials and Methods (Section 2.8). For panels B-D, raw gene counts for UGT2B10, UGT2B15, and UGT2B17 were obtained from the GSE130970 dataset, and fold-change in relative expression was calculated directly.

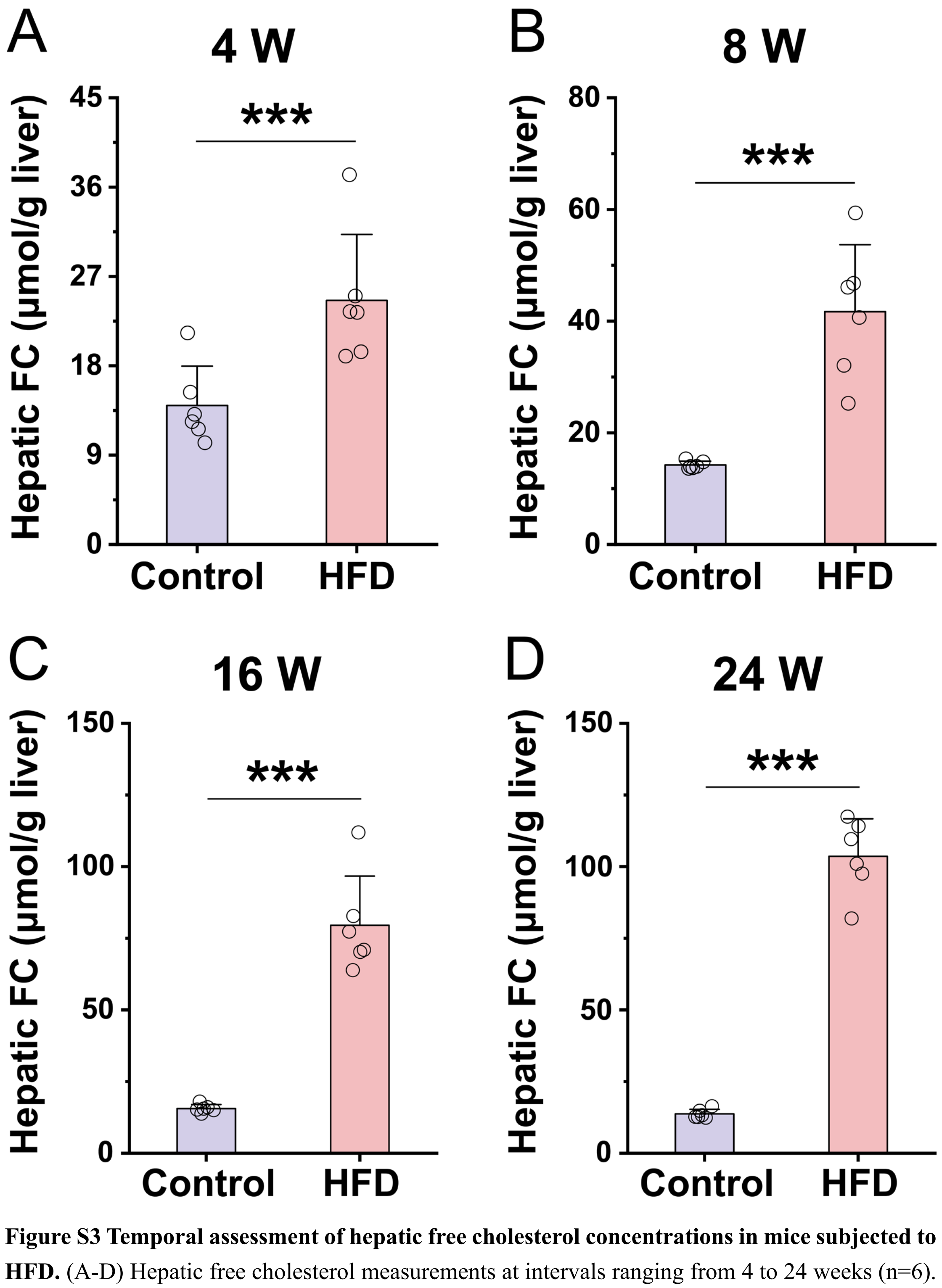

Figure S3 Temporal assessment of hepatic free cholesterol concentrations in mice subjected to HFD. (A-D) Hepatic free cholesterol measurements at intervals ranging from 4 to 24 weeks (n=6). Data are presented as mean ± SD. Statistical significance was determined by unpaired two-tailed Student's t-test. ***, P<0.001.

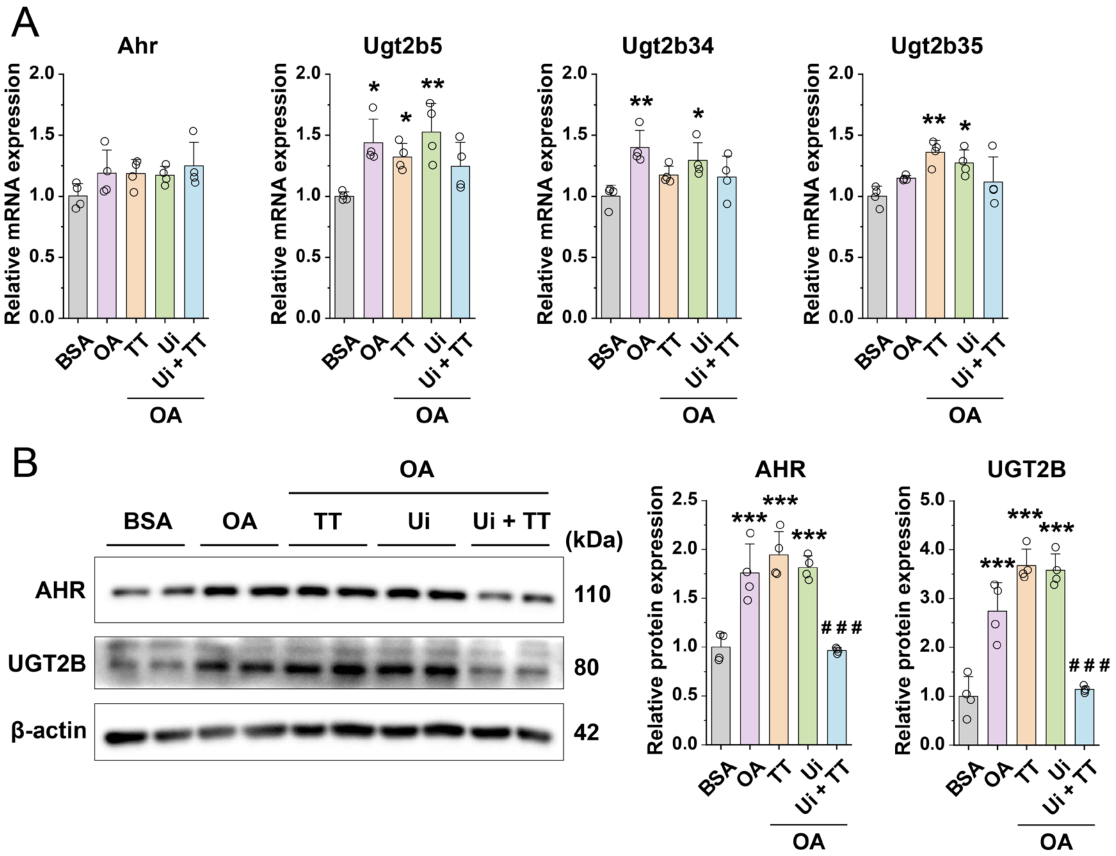

Figure S4 Impact of oleic acid (OA) and testosterone treatments on the testosterone inactivation pathway within an OA-induced cellular model. (A) Gene expression levels of Ahr and Ugt2b isoforms (Ugt2b5, Ugt2b34, Ugt2b35) (n=4). (B) Protein expression of AHR and UGT2B along with their quantification (n=4). Note: The observed protein band of UGT2B was detected using a human UGT2B10 antibody, which detected a band near 80 kDa. This band likely corresponds to the mouse Ugt2b protein. Data are presented as mean ± SD. Statistical significance was determined by one-way ANOVA followed by Tukey's HSD post-hoc test. The pre-specified primary contrast between the OA and Ui+TT groups is highlighted. For the comparison between the BSA group and other groups: *, $P<0.05$; **, $P<0.01$; ***, $P<0.001$. For the comparison between the OA group and other groups: #, $P<0.05$; ##, $P<0.01$; ###, $P<0.001$.

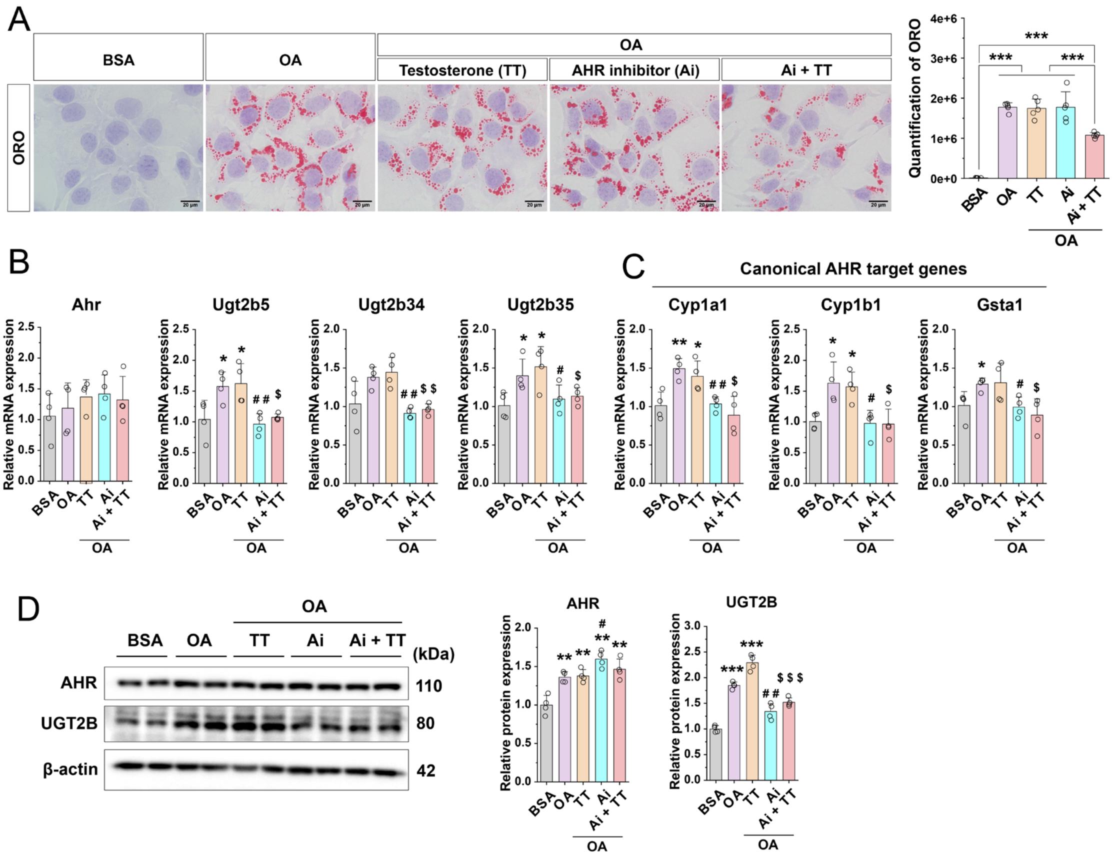

Figure S5. AHR is required for OA-induced UGT2B upregulation and consequent testosterone inactivation in AML-12 cells. (A) Representative Oil Red O (ORO) staining and quantitative analysis of lipid accumulation in AML 12 cells (n=5). (B) Relative mRNA expression of Ahr and UGT2B family genes in AML 12 cells from each treatment group (n=4). (C) Relative mRNA expression of canonical AHR target genes (n=4). (D) Representative WB and quantitative analysis of AHR and UGT2B protein levels (n=4). Note: The observed protein band of UGT2B was detected using a human UGT2B10 antibody, which detected a band near 80 kDa. This band likely corresponds to the mouse Ugt2b protein. Data are presented as mean ± SD. Statistical significance was determined by one-way ANOVA followed by Tukey's HSD post-hoc test. The pre-specified primary contrast between the Ai and Ai+TT groups is highlighted. For the comparison between the BSA group and other groups: *, P<0.05; **, P<0.01; ***, P<0.001. For the comparison between the OA group and Ai group: #, P<0.05; ##, P<0.01; ###, P<0.001. For the comparison between the Ai group and Ai + TT group: $, P<0.05; $$, P<0.01.
